# Study of Different Cultivated Plants Rhizosphere Soil Fungi-Mediated Pectinase: Insights into Production, Optimization, Purification, Biocompatibility, and Application

**DOI:** 10.1007/s00248-024-02474-0

**Published:** 2025-01-06

**Authors:** Mai Ali Mwaheb, Basant Mohamed Abd El-Aziz, Basma T. Abd-Elhalim, Nabil Abo El-Kassim, Tharwat E. E. Radwan

**Affiliations:** 1 Department of Botany, Faculty of Science, Fayoum University, Fayoum, 63511 Egypt; 2 Department of Agricultural Microbiology, Faculty of Agriculture, Ain Shams University, Hadayek Shoubra, P.O. Box 68, Cairo, 11241 Egypt

**Keywords:** Bioscouring, Human skin cell line (HFb-4), Microorganisms, Pectinase, Rhizosphere

## Abstract

Microorganisms are preferred as an enzyme source due to their short lifespan, high production rate, affordability, and absence of harmful chemicals in enzymes generated from plant and animal sources. Fungi communities are biological factories for many bioactive compounds such as the important industrial enzyme pectinase. The current study dealt with production, optimization, purification, biocompatibility, and application of fungal pectinase obtained from five plant rhizospheres (banana, jarawa, lemon, tomato, and wheat) at Fayoum Governorate, Egypt. The highest pectinase degrading index (PDI) was scored for FB5, FJ2, and FW1 isolates. Pectinase production was also examined quantitively and the highest output of 1603.67, 1311.22, and 1264.83 U/ml was gained by FB5, FJ1, and FW1 fungal isolates, respectively. The most active pectinase-producing fungi were identified as *Aspergillus niveus* strain AUMC1624, *A. niger* strain AUMC16245, and *A. brasiliensis* strain AUMC16244, respectively. For pectinase production optimization, one factor at a time (OFAT) protocol was applied and revealed that *A. niger*, *A. niveus*, and *A. brasiliensis* reached maximum pectinase levels at 1% pectin after 5, 7, and 7 days, at 40, 45, and 45 °C, respectively. Obtained pectinases were partially purified using ammonium sulfate precipitation (ASP) and organic solvent precipitation (OSP) methods. The highest activity using the ASP method scored at 40–60% saturation with *A. niger*. The thermostability characterization of *A. niger* pectinase was reached with relative activities of 61.7, 69.0, 99.9, 91.3, and 90.6% at temperatures ranging between 30 and 70 °C. pH optimized at pH 5–7. The enzyme’s molecular weight was approximately 35 kDa. The GC-mass analysis of pectinase end products included acetic acid ethyl ester, hexadecane carbonsaure methylase, and hexadecenoic acid. The biocompatibility was examined using a human skin cell line (HFb-4) for the first time, with a minimal half concentration (IC_50_) of 151.86 ± 0.76 U/ml. The biocompatible pectinase was applied as a clothes bioscouring agent with different concentrations of 1893.52 U/ml achieving the highest bioscouring with 20.0%.

**Supplementary Information:**

The online version contains supplementary material available at 10.1007/s00248-024-02474-0.

## Introduction

Enzymes that are produced by fungi, bacteria, animals, and plants are crucial biocatalysts for a range of commercial and biotechnological uses. Compared to chemical catalysts, they can function in a variety of challenging conditions. As a result, an assortment of microbial enzymes are employed in biotechnology and other fields. Because of their short lifespan, high rate of productivity, affordability, and lack of dangerous compounds, which are present in enzyme derived from plant and animal sources, microorganisms are favored as a source of enzymes [1]. Among the most significant industrial enzymes are pectinases. Pectinase is a crucial enzyme that plays a significant role in both the breakdown of pectin in plant cell walls and is a key component of those walls [1, 2]. Pectin is a complex glycosidic macromolecule with a large molecular weight that is acidic and negatively charged. Pectinase is the most widely produced enzyme worldwide, making up around 10% of the global market. It breaks down polygalacturonic acid by cleaving its α−1, 4-glycosidic bond, releasing free galacturonic acid, and reducing-sugar end-groups. Yeasts, fungi, and bacteria are among the many organisms that can produce pectinolytic enzymes [2, 3]. Approximately 25% of all enzymes produced and sold worldwide are microbial pectinases. The production of pectinases by actinomycetes and fungus *Aspergillus* sp. has been the subject of several investigations [4, 5]. Pectinases may be divided into three groups: protopectins, pectin esterases, and depolymerizing enzymes. Alpha (1,4) glycosidic links in the D-galacturonic acid moiety of pectic compounds are hydrolytically cleaved by the depolymerizing enzyme polygalacturonase [6]. It catalyzes hydrolytic cleavage at the nonreducing end of the substrate, which might sometimes result in mono-galacturonate or digalacturonate. Pectinase may be made in a number of methods, such as submerged fermentation and solid state fermentation, both of which rely on the strain’s optimal pH and temperature [7]. Researchers are interested in pectinolytic enzymes synthesized by microorganisms because they may be used as biological catalysts in various industrial processes [8]. The three primary industrial applications of pectinases are in fruit juice extraction, clarification, and textiles processing. Pectin is primarily responsible for the turbidity and viscosity of apple juice. Fruit juices are clarified by the combined action of pectinases and amylases, which can cut the filtering time in half [9]. Humans can benefit from pectinases in a number of ways, including enhanced absorption of plant-based diets and the development of balanced gut microbiota [10]. Also, pectinase enzymes are used in the textile industry as bioscouring agents to enhance the textiles’ quality but it is still a concern that it can remain as traces on the textile materials used and causes skin irritation, so it is important to control their effects on humans by testing its safety and biocompatibility [11]. So, the current work demonstrates fungi producing pectinases using one factor at a time optimization, characterization, purification, and biocompatibility using skin normal cells for the first time to be investigated. Purified pectinase is applied as a bioscouring agent in the textiles industry as one of the sectors representing pectinase utilizing sectors.

## Materials and Methods

### Samples Collection

Different rhizosphere soil samples were taken from cultivated soils from the upper 10.0 cm of the soil surface [12, 13], and each sample weighed roughly 0.5 kg. Samples were gathered from five different crops rhizosphere of banana (*Musa acuminata*), jarawa (*Glossonema varians*), lemon (*Citrus aurantiifolia*), tomato (*Solanum lycopersicum*), and wheat (*Triticum aestivum* L.) from various fields in different Fayoum Governorate centers, including Al Fayoum Center (29.3565″N, 30.6200″E), Sinnuris Center (29.407521″ N 30.8663985″ E), Tamiya Center (29° 28′ 40.75″N, 30° 57′ 41.92″E), and Ibsheway Center (29° 21′ 25.25″N 30° 40′ 49.94″E), Egypt, respectively. Soil samples were immediately transferred to the microbiology laboratory at the Microbiology Department, Science Faculty, Fayoum University for further study and testing. All samples were stored at 4 °C.

### Isolation of Soil Rhizosphere Fungi

According to serial dilutions technique [14], 35 g of each rhizosphere soil sample was weighed and blended in 315.0 ml of sterilized distilled water (SDW). The samples were mixed well for 15 min using a vortex mixer (Witeg labortechnik, Germany) until they were homogenized, and then serially diluted 10^−1^ up to 10^−6^. Approximately 20–25 ml of potato dextrose agar (PDA) medium [12] was loaded onto sterile petri plates, and approximately 7.0 ml was poured into test tubes set at a slant position in the microbiological hood (Class II A2 Biological Safety Cabinet, Esco Micro, Singapore) and left to solidify at room temperature in order to make slants to preserve the selected isolates. From the serial dilutions, 0.1 ml was inoculated on the prepared PDA plates by the spread plate technique and inversely incubated (biochemical incubator CBI Taisite, USA) at 28 °C for 3–5 days. To obtain pure culture, the distinct each fungal colony was sub-cultured repeatedly.

### Qualitative Screening of Pectinase-Producing Fungi

Isolated fungi was inoculated of 0.1 ml spore suspension (10^7^ spores/ml) in prepared pectinase screening agar (PSA) medium [12] and incubated for 48h for identification of high yield pectinase producer. The petri dishes were incubated in an inverted position. After incubation, the pectinase-producing isolates that showed clear zone around the margins of the colony were detected by flooding the dishes with 50 mM I_2_ solution. The colony diameter and clear zone diameter were measured in centimeters (cm) for the pectinolytic index (PI) as follow [12]:

1 $$Pectinolytic\;Index \left(PI\right)=\frac{\left(clear\;zone\;diameter- Colony\;diameter \right) }{Colony\;diameter } *100$$

The positive fungal pectinase producers were subcultured at monthly intervals and preserved on PDA slants at 4 °C. For identification and further enzyme investigations tests, long-term storage was kept at − 20 °C in PDA medium and 30% glycerol [14].

### Production of Pectinase Using Submerged Fermentation Technique (SmF) (Quantitative Estimation)

The most potent pectinase selected fungi was inoculated with 5.0 ml (10^7^ spores/ml) in prepared PSF medium and agitated in shaker incubator (Lab. Companion SI 600R, UK) at 200 rpm for 5 days at 28 °C. Every 24 h of time interval, 10 ml fermented medium samples was taken and filtered through Whatman filter paper no. 1. This filtrate was considered a crude enzyme and was used for the analysis and to determine pectinase activity. The crude enzyme was then kept at 4 °C for storage [12].

### Phenotypic and Genotypic Identification of Pectinase-Producing Fungi

The most potent pectinase-producing fungal isolates were identified based on their morphology, mycelia structure, and spore formation [15]. The genomic 18S rRNA was isolated utilizing the Big-Dye terminator kit ABI 310 Genetic Analyzer (Applied Biosystems, USA); the purified PCR product was sequenced [16]. To determine which fungi were closest to one other, partial 18S rRNA sequence data were aligned and examined. Using the BLASTN tool, which is accessible at the National Center for Biotechnology Information (NCBI, 2024), the unknown query 18S rRNA nucleotide sequence was compared to nucleotide databases and retrieved from the GenBank database. Following that, the Clustal Omega method was used to create multiple sequence alignment for these homologous sequences. The neighbor joining approach was then used to create a phylogenetic tree.

### One Factor at a Time (OFAT) Optimization for Pectinase Production

The present investigation employed the one factor at a time (OFAT) technique to examine and optimize the production of fungal pectinase [17]. Several nutritional factors (suitable substrate (pectin) concentration and nitrogen sources) and environmental factors (incubation time, pH, and temperature) were taken into consideration for pectinase optimization. To find out the optimum level of pectin, each fungal culture was inoculated into a PSF medium at five different concentrations of 0.25, 0.5, 0.75, 1.0, and 2%. The other factors remained the same as subsequently mentioned previously which are incubated for 5 days at 30 °C. The enzyme production was investigated for 12 days, at specific intervals (every 24 h) starting from the third-day fermentation. A sample was taken from each pectin broth flask and underwent filtration as mentioned previously. The optimum temperature was assayed by incubation of the inoculated pectin broth at various temperatures ranging between 25 and 55 °C with incubation under the proper time and shaking circumstances of incubation. Each culture fermented medium was filtered through Whatman filter paper no. 1 and the resulting clear filtrate (known as a crude enzyme) was used for pectinase activity estimation.

### Determination of Pectinase Activity Using 3,5-Dinitrosalicylic Acid (DNS) Technique

The quantity of pectinase released in the fermented broth was measured by 3,5-dinitrosalicylic acid (DNS) technique [18]. The amount of reducing end products present and the number of glycosidic bonds the enzyme cleaves determine how intense the color is generated by this test. To express the pectinase activity into an enzyme unit, the D-galacturonic acid standard curve was used at 540 nm to measure the absorbance using a spectrophotometer (AE-UV90, A7E LAB, UK). Determine the quantity of D-galacturonic acid (mM) released based on the enzyme’s action related to the enzyme activity in U/ml using the following equation:

2 $$Pectinase\;Activity\;(U/ml) = \frac{Conc. \left(mM\right)*1000}{Incubation\;time\;(mins)}*dilution\;factor$$

Dilution factor is the dilution of the original enzyme preparation, and it could be calculated by using the following equation:

3 $$Dilution\;factor= \frac{final\;solution\;volume}{sample\;aliquot\;volume}$$

4 $$Concentration\;of\;Enzyme \left(mM\right)=\frac{(OD 540 -0.0239)}{0.0746}$$

The amount of pectinase that catalyzes the synthesis of 1 µmol galacturonic acid under test conditions was defined as one unit of the enzyme [12, 18].

### Pectinase Partial Purification

#### Ammonium Sulfate Precipitation (ASP) Method

Various concentrations of solid ammonium sulfate (NH_4_)_2_SO_4_ were added to a final volume of 1 L pectinase crude enzyme to achieve 0–80% saturation at 4 °C. The supernatant was removed and the pellets collected from every phase of ammonium sulfate saturation were individually dissolved in 1 ml of pH 4.7 acetate buffer. The excess ammonium sulfate was then removed by dialyzing the pellets using a Spectra/PorR, VWR 2003 dialysis bag against the same buffer for a whole night at 4 °C and twice changing the washing buffer. Prior to and during the dialysis of enzymes precipitated by ammonium sulfate, total protein and enzyme activity were measured [19, 20].

#### Organic Solvents Precipitation (OSP) Method

One milliliter of the enzyme protein solution was combined with 4 and 9 ml of cooled acetone and absolute cold ethanol, respectively, for a minimum of 60–90 min at − 20 °C. After that, it was centrifuged for 10 min at 4 °C at 10,000 rpm. After removing the supernatant, the pellets were gathered and allowed to air dry in order to eliminate any solvent impurities [20–22]. They were then dissolved in acetate buffer (pH 4.7) and dialyzed in accordance with the previously stated procedure [23].

### Total Protein Determination

The total protein was determined using bovine serum albumin (BSA) as standard solution [24]. Total protein was calculated to estimate the specific activity of enzyme as follows:

5 $$Specific\;activity (U / mg\;protien) = (Activity\;of\;enzyme\;in\;the\;sample\;(U/ml) )/Total\;protien\;(mg/ml)$$

### Characterization of Pectinase Enzyme

The effect of temperature, pH, and incubation time on enzyme behavior was studied and the relative activity compared to the enzyme activity was calculated. The ideal temperature for pectinase was monitored for 30 min at temperatures between 40 and 100 °C. The relative pectinase activity (%) was then calculated using standard assay conditions. The untreated enzyme’s activity (control) was assumed to be 100% [20, 21, 25].

6 $$\% Relative\;Activity = (Activity\;of\;enzyme\;(U/ml) )/(Initial\;enzyme\;activity\;(U/ml))\;X100$$

The partial purified enzyme solution was incubated at different temperatures ranging from 40 to 100 °C for 15 to 60 min in order to determine the heat stability, or Q10, of the pectinase enzyme. Each treatment was then ended by exposure to ice-cold water, and the relative activity was determined at the optimal temperature in accordance with the standard assay conditions [20, 21, 26]. Also, pectinase activity was measured using a range of pH buffers, including citrate buffer (pH 3.4, 4.4, and 5.4), sodium phosphate buffer (pH 6.4–7.4), and borate buffer (pH 9.0), in order to establish the ideal pH. The enzyme solution was combined with the aforementioned solutions for the pH stability investigation, and it was then incubated for 15 to 60 min. The relative pectinase activity was expressed as a percentage [20, 21].

### Gas Chromatography (GC–MS) of Pectinase Metabolic Profile

The internal standards, 100.0 µl methanol, 50.0 µl ribitol, 50.0 µl 3,5-dichloro-4-hydroxybenzoic acid, and 100.0 µl nonadecanoic acid, were combined with a 10-ml pectinase-pectin combination mixture (enzyme–substrate-complex). After 10 s of mixing in vortex, the samples were incubated at 70 °C for 30 min [27]. After that, 500.0 µl of chloroform and 300.0 µl of distilled water were added. The mixture was cooled to room temperature and centrifuged (10 min, 13,000 rpm) for 10 s. The preparation of the polar and nonpolar fractions followed the directions provided by. Following their extraction, the fractions underwent GC–MS analysis utilizing a 7890A gas chromatograph connected to a 5975C Agilent Technologies inert XL EI/CI MSD mass detector (Agilent Technologies, Santa Clara, CA 95051, USA) operating at 70 eV at Cairo University Research Park (CURP), Giza, Egypt.

### Pectinase SDS Page Analysis

The following procedure was used to create the separating gel (15%); a 5.0 ml (29.2% acrylamide and 0.8% bis-acrylamide), 2.5 ml 1.5M tris pH 8.8, 100µl of 10% SDS, 100µl of 10% APS, and 100µl of Tetramethylethylenediamine (TEMED) were combined with 2.4 ml of distilled water (DW) [28]. Subsequently, the stacking gel (4%) was made as follows: 100 µl 10% SDS, 100 µl 10% APS, 100 µl TEMED, 1.3 ml (29.2% acrylamide and 0.8% bis-acrylamide), and 2.5 ml 0.5M tris pH 6.8 were all included in the sample. After preparing the samples (300 µl saline solution with 100 mg sample), they were centrifuged for 5 min at 10,000 rpm while being cooled to 4 °C. From the prior mixture, 500 µl of acetone was added and left overnight at − 20 °C. The mixture was then centrifuged for 5 min at 4 °C at 10,000 rpm. Sample buffer (10% SDS, 20% glycerol, 0.2 M tris pH6.8, 10 mM beta-mercaptoethanol, and 0.05% bromophenol blue) should be mixed with one volume of the sample in four volumes. The mixture should then be heated for 5 min at 95 °C. A 25 mM Tris–HCl, 200 mM glycine, and 0.1% (W/V) sodium dodecyl sulfate (SDS) were added to create the running buffer, which then started at 80 V for 4 h. Following the completion of the run, the gel was gently stirred for 20 min with a staining solution consisting of 50% DW, 40% methanol, 10% glacial acetic acid, and 0.1% Coomassie brilliant blue. Lastly, destain the gel that was produced using a destaining solution that contains 10% glacial acetic acid, 40% methanol, and 50% DW. The analysis was performed at Cairo University Research Park (CURP), Giza, Egypt.

### Biocompatibility of Pectinase Using 3-(4,5-Dimethylthiazol-2-yl)−2,5-diphenyl-2H-tetrazolium Bromide (MTT) Assay

Using human skin cell line (HFb-4), the biocompatibility of partially purified pectinase was determined at the Science Way for Scientific Researches and Consultations firm located in Cairo, Egypt. To create a full monolayer sheet, 1 × 10^5^ cells/ml (100 µl/well) was injected and cultured at 37 °C for 24 h using a 96-well tissue culture plate. Once a confluent sheet of cells had formed, the growth material was removed from the 96-well microtiter plates and the cell monolayer was washed twice with wash media. The sample analyzed was diluted twice and placed in Roswell Park Memorial Institute Medium (RPMI) medium containing 2% serum (maintenance medium). Each dilution, ranging from 0.1 to 4 ml, was examined in distinct wells, with three wells serving as controls and receiving just maintenance media. The plate was then inspected after being incubated at 37 °C. We examined the cells for any outward signs of toxicity. In phosphate buffer solution (PBS) (5 mg/ml), the MTT solution was produced (Bio Basic Inc., Canada). To fully mix the MTT into the medium, a 20-µl MTT solution was poured to each well and the shaking table was set at 150 rpm for 5 min. Incubation was done for 4 h at 37 °C with 5% CO_2_ to facilitate the metabolism of MTT. After discarding the extra medium, formazan (a metabolic product of MTT) was once again suspended in 200 µl of dimethyl sulfoxide (DMSO). Next, position on a shaking table and shake at 150 rpm for 5 min in order to fully combine the formazan and solvent. At 560 nm, the optical density was measured, and at 620 nm, the background was subtracted [29].

### Application of Pectinase as Bioscouring Agent

The partially purified enzyme was applied as bioscouring (excess pectin removing) agent in textile industry. In the current study, a stiff piece of black cloth that had been soaked with 1% pectin was employed. A piece of cloth measuring 6 cm × 6 cm was weighed. Using the double dilution method, various concentrations of the described partially purified pectinase (1, 1/2, 1/4, 1/8, 1/16, 1/32, 1/64) were created in the ideal buffer solution (pH) and temperature for 45 min. After being cleaned with tap water, the fabric strip was dried in the oven. Following drying, the cloth strip was weighed once again, and the percentage of pectin removed was computed using the following equation [30]:

7 $$\%\;Removal\;of\;pectin\;(bioscouring) = (Weight\;of\;pectin\;removed\;by\;pectinase\;/\;Total\;pectin\;present\;on\;the\;fabric\;strip)\;x\;100$$

### Statistical Analysis

The designs of experiments, analysis of data, the statistical differences and significance (*p* < *0.05*), the figure representations, and the optimization were carried out by OriginPro 2022 statistical package software version V.9.9.0.225 (SR1) (OriginLab Corporation, Northampton, USA) using analysis of variance (ANOVA) and means of difference by Duncan analysis [31]. All experiments were performed in three replicates and the results were presented as means ± SE.

## Results

### Isolation of Soil Pectinase-Producing Fungi

**Fig. 1:**
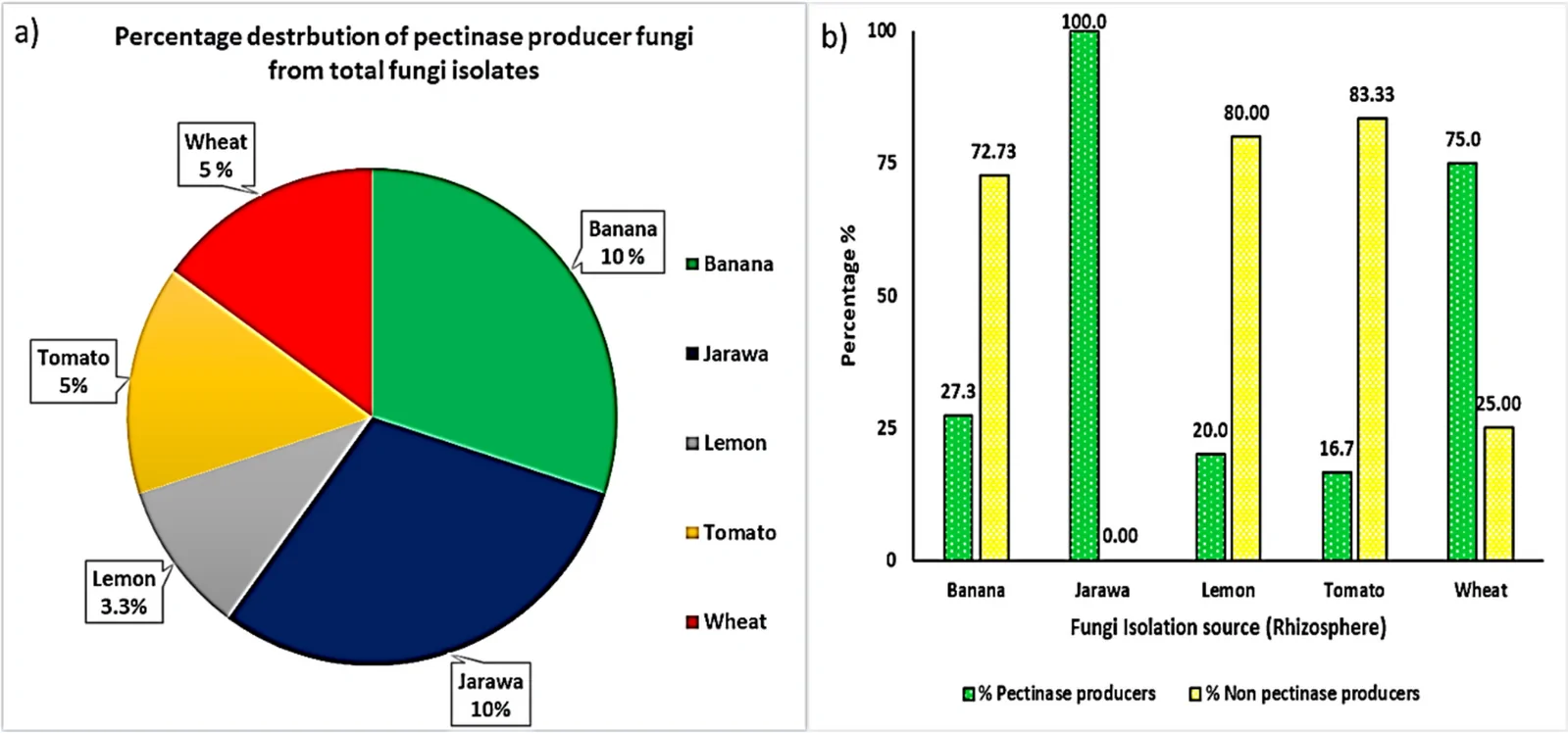
**a**) Percentage distribution of fungal isolates obtained from different plants rhizosphere, **b**) percentage distribution of positive pectinase fungal isolates from total isolates of different plants rhizosphere

In the present study, 60 fungi isolates were obtained from five plants rhizospheres, namely Banana (*Musa acuminata*), Jarawa (*Glossonema varians*), lemon (*Citrus aurantiifolia*), tomato (*Solanum lycopersicum*), and wheat (*Triticum aestivum* L.). The fungal isolates were distributed among the sources as follows: 22, 6, 10, 18, and 14 fungal isolates from banana, jarawa, lemon, tomato, and wheat, respectively, as illustrated in Table (S1) and Fig. 1a. The number distribution of the pectinase and non-pectinase-producing fungi varied among the isolation sources as shown in Fig. 1b. The positive pectinase fungal isolate percentage out of all total isolates of each isolation source was 10%, 10%, 3.3%, 5%, and 5% for banana, jarawa, lemon, tomato, and wheat, respectively (Fig. 1b).

### Screening and Selection of Pectinase-Producing Fungi

**Fig. 2:**
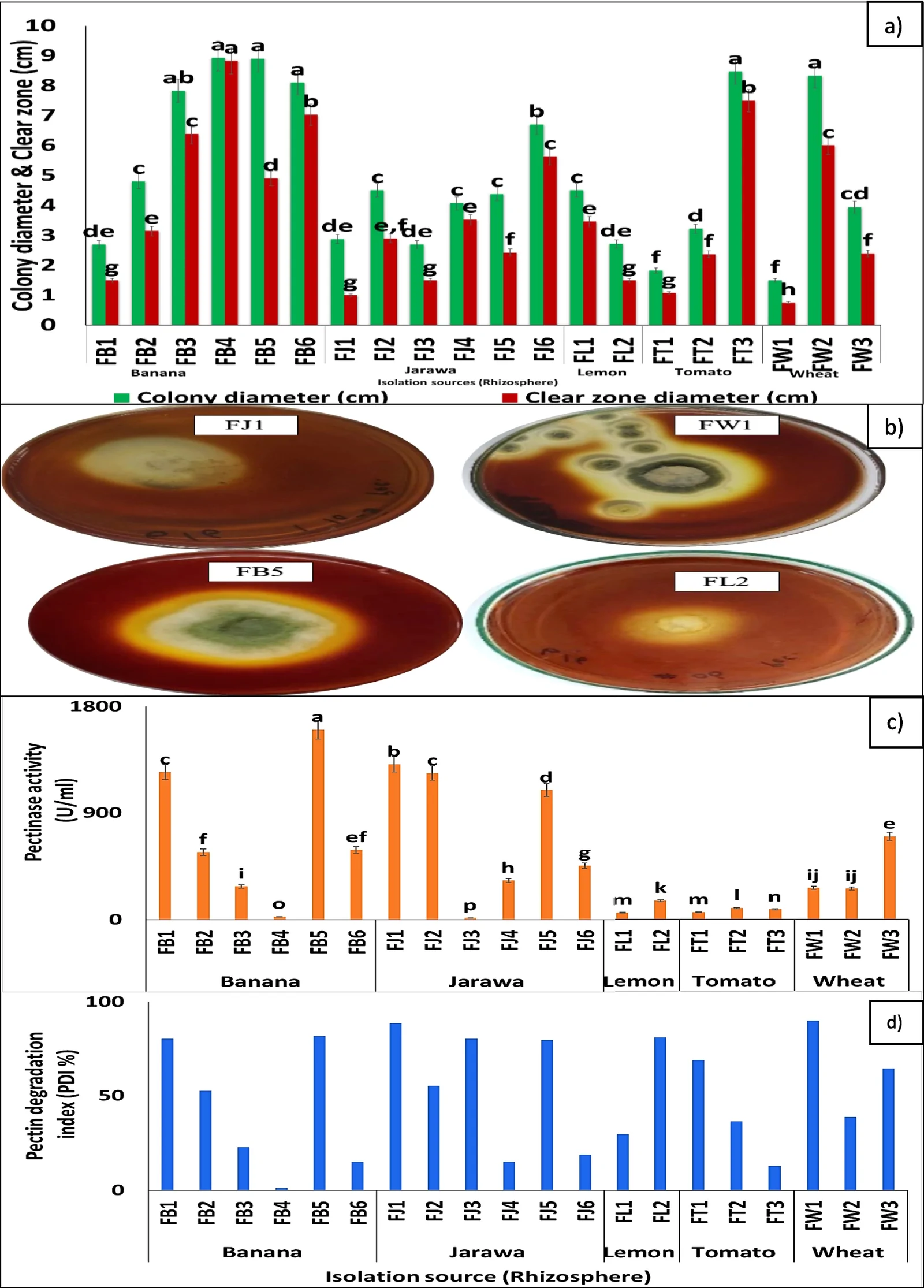
**a**) Colony and clear zone diameter for pectinase-producing fungi, **b**) qualitative screening of pectinase, **c**) pectinase activity, and **d**) pectin degradation index (PDI%) of selected pectinase-producing fungi at 28 °C for 72 h on PSA (**a** and **b**) and PSF (**c** and **d**) medium. Values followed by the same letter are not significantly different, according to Duncan at a 5% level

In the basis of pectinolytic clear zone and degradation index, the most active fungi was selected. Data recorded in Table S2 and illustrated in Fig. 2a and b clearly show that clear halo-zone diameter and PDI ranged from 8.83 to 0.75 cm and 88.3 to 1.13%, respectively. The statistical analysis proved that fungal isolates FB5, FJ2, and FW1 gave the highest values of PDI, while the lowest PDI values were recorded for FB6, FJ4, and FT3. The pectinase production and pectinase degradation index (PDI%) by the isolated fungi ranged from 14.23 to 1603.67 U/ml and 1.13 to 90% as viewed in Table (S2) and Fig. 2c and d, respectively. The fungal isolate FB5, FJ1, and FW1 were the pioneer pectinase producers with 1603.67, 1311.22, and 1264.83 U/ml, respectively.

### Phenotypic and Genotypic Identification of the Most Pectin-Producing Fungi

**Fig. 3:**
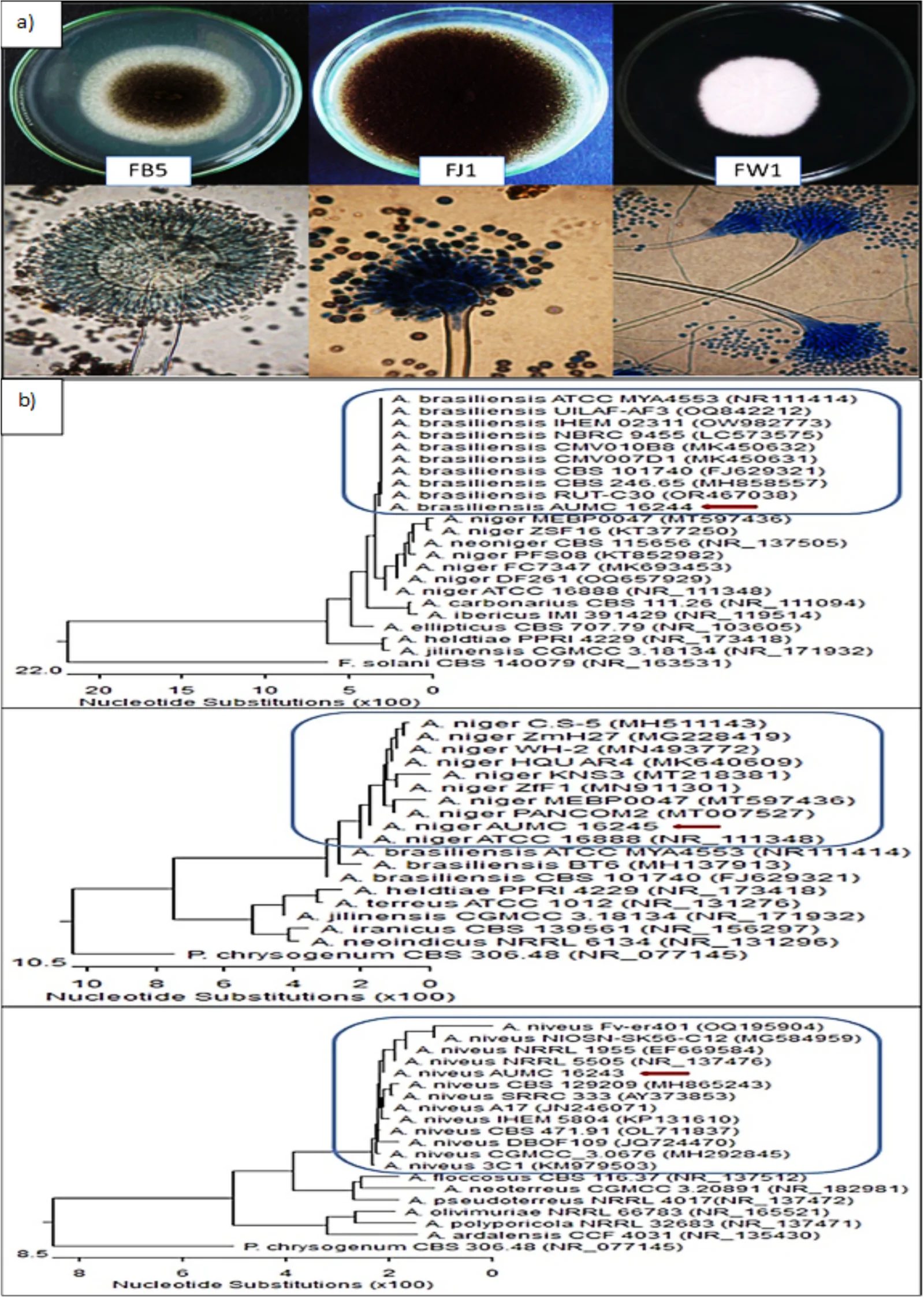
**a**) Morphological characterization of pectinase-producing fungal isolates cultivated at 28 °C for 72 h on PDA medium from the left *A. brasiliensis*, *A. niger*, and *A. niveus*, respectively. **b**) Phylogenetic tree of fungal isolates based on ITS sequences of 18S rRNA

As shown in Fig. 3a, isolate FB5 has a cottony appearance, initially white, then gradually turns black. It forms filamentous hyphae as small plants, with conidial heads radiated with conidiogenous cells biseriate. Conidia are brown to black, and the conidiophores are protrusions from a septate and hyaline hyphae belonging to *Aspergillus brasiliensis*, while the isolate FJ1 has a cottony appearance, initially white, then black, and is made up of felt-like conidiophores. Macroscopic observations of *Aspergillus niger* reveal initial white growth, but it changes to black after a few days, producing conidial spores. On the other hand, isolate FW1 has smooth colored conidiophores and conidia, with conidiophore stipes smooth walled and hyaline up to 1000 μm long. Conidia are spherical, hyaline, and 2–2.5 μm thick, belonging to *A. niveus*. The phylogenetic tree results found that the *A. niveus* (isolate FB5) showed 99.79–100% identity with several strains of the same species and took a GenBank accession number of *A. niveus* strain AUMC16243. The *A. brasiliensis* (isolate FJ1) showed 98.61–100% identity coverage and took a GenBank accession number of *A. brasiliensis* strain AUMC16244, while *A. niger* (isolate FW1) showed 100% coverage and it was deposited at GenBank with accession number of *A. niger* strain AUMC16245 (Fig. 3b).

### One Factor at a Time (OFAT) Optimization of Pectinase Production

**Fig. 4:**
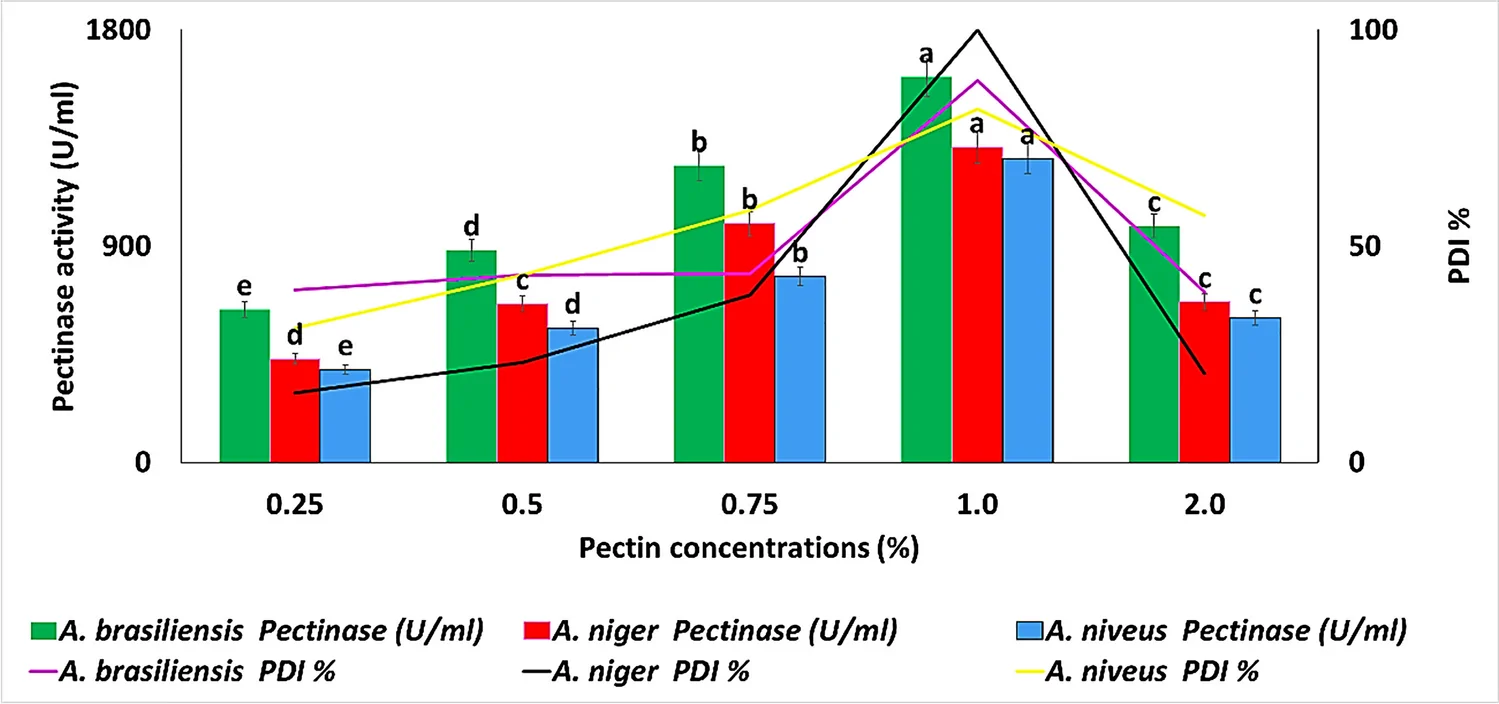
Effect of different concentrations of pectin on pectinase production on PMA medium by different *Aspergillus* spp. on PSA medium at 28 °C for 72 h. Values followed by the same letter are not significantly different, according to Duncan at a 5% level

**Fig. 5:**
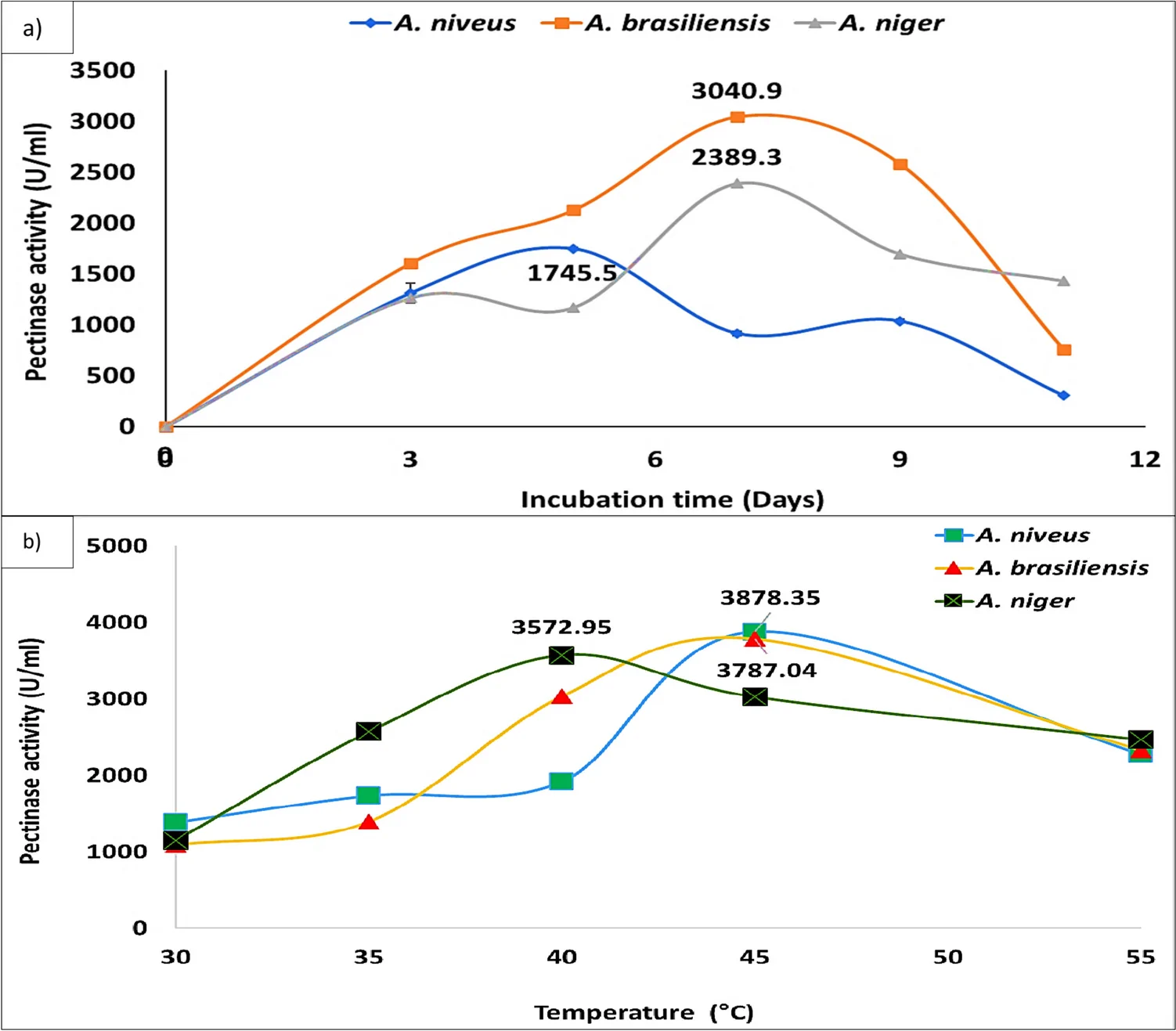
**a**) Time course of pectinase production by different *Aspergillus* spp. on PMA medium at 28 °C. **b**) Incubation temperature effect on pectinase production by different *Aspergillus* spp. on PMA medium with the suitable incubation time

Data illustrated in Fig. 4 clearly show that there was a gradual increase in the pectin concentration that increased after the saturation took place; it decreased also gradually. It was found that the PDI% and pectinase activity of *A. brasiliensis*, *A. niger*, and *A. niveus* reached the maximum level at 1% pectin using PSM and PMA media, respectively, and any increase or decrease in pectin concentration led to minimization of their values. The most active fungi among the three tested fungi was *A. brasiliensis*, at 28 °C for 72 h with PDI and pectinase activity of 88.3% and 1603 U/ml, respectively. The results (Fig. 5a) revealed that *A. brasiliensis*, *A. niger*, *and A. niveus* highly produce pectinase after 5, 7, and 7 days of incubation periods. The most appropriate temperature on pectinase production by *A. brasiliensis*, *A. niger*, and *A. niveus*, was studied using different incubation temperatures that ranged between 30 and 55 °C at the suitable incubation period for each fungus. The results showed that *A. niger*, *A. brasiliensis*, and *A. niveus* highly produce pectinase after 40, 45, and 45 °C, with 3787.04, 3878.35, and 3572.95 U/ml, respectively (Fig. 5b).

### Pectinase Partial Purification

The concentration of ammonium sulfate that was used in protein precipitation varied in the range of 0–20%, 20–40%, or 40–60% (w/v). According to the ammonium sulfate fractionation, the maximum relative activity of pectinase was at the concentration of ammonium sulfate of 40–60% saturation for *A. niger* pectinase, with specific activity of 48.13 related to the crude enzyme. The enzymes were partially purified by organic solvents (ethanol and acetone) and the results are summarized in Table (S3). In the case of using ethanol, *A. brasiliensis* was the highest with relative activity of 92.14%, while for acetone, *A. niveus* was the highest with relative activity of 94.4%.

### Characterization of Partially Purified Pectinase

#### Effect of Temperature on Pectinase Activity and Stability

**Fig. 6:**
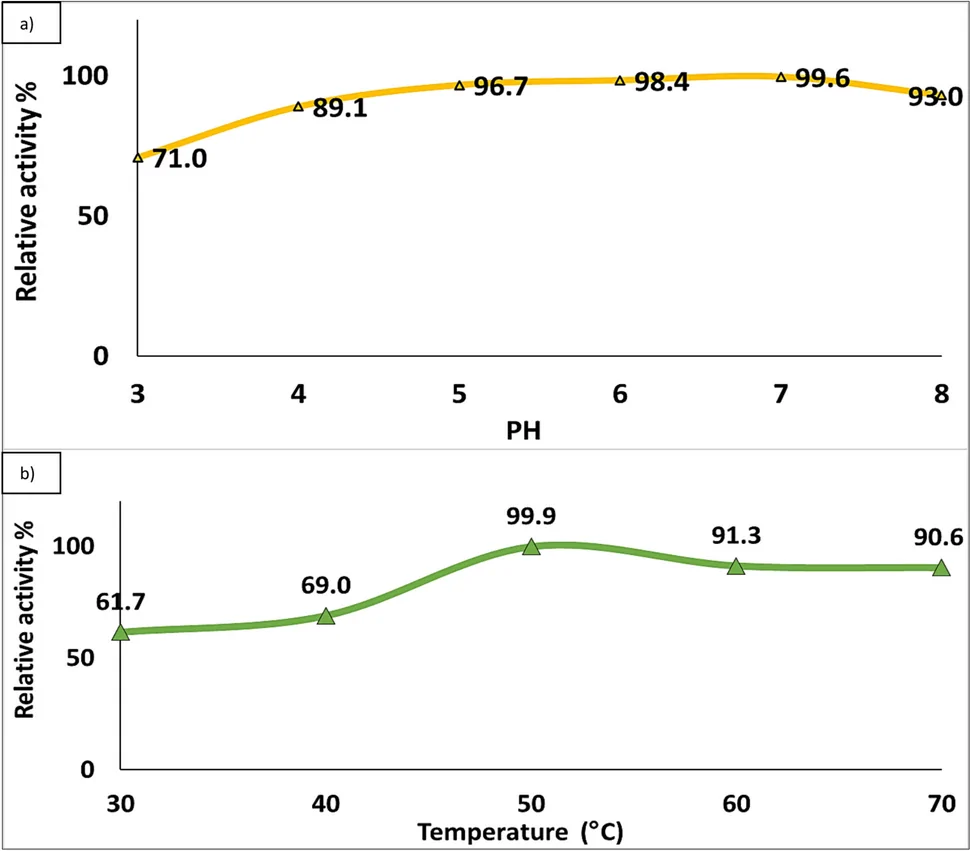
**a**) Effect of temperature. **b**) Effect of pH on the activity and stability of pectinase by *A. niger*

Incubation temperature affects the activity of *A. niger* pectinase. The activity was determined by carrying out the assay at several temperatures between 30 and 70 °C. Results are presented in Fig. 6a, showing that the enzyme activity increased by increasing temperature to 50 °C, then decreased. As the enzyme obtained relative activities of 61.7, 69.0, 99.9, 91.3, and 90.6% when treated with temperatures of 30, 40, 50, 60, and 70 °C, the temperature coefficient Q10, which is the factor used to evaluate the velocity of enzyme reaction when temperature is raised by 10 °C, is typically used to describe the influence of temperature on enzyme reaction [32]. Enzyme reactions typically have a temperature coefficient between 1 and 2. Results recorded in Table (S4) clearly show that Q10 of pectinase was 1.12, 1.45 between 30–40 °C and 40–50 °C, whereas the Q10 value was 0.91, 50–60 °C and between 60 and 70 °C.

#### Effect of Temperature on Pectinase Activity and Stability

It was observed that enzymatic activity has a broad pH range from 3.0 to 8.0. Results presented in Fig. 6b showed that *A. niger* pectinase exhibited high activities at pH values between 5 and 7, then decreased with raising or lowering pH than the optimum levels. The optimum pH 7.0 achieved the maximal activity for pectinase with 99.6% relative activity.

#### GC–MS for Pectinase Enzyme End Product Profile

Based on Table (S5), the GC–MS analysis of pectinase enzyme end products confirmed the presence of acetic acid ethyl ester, hexadecane carbonsaeuremethylese, hexadecenoic acid, 9-octadecenoic acid (z), cis-vaccenic acid, octadecanoic acid, oleic acid, glycidyl palmitate, ethyl iso-alcoholate, 2,3-dihydroxypropyl ester, and arabinitol pentaacetate as the main end product of pectinase reaction.

## SDS-Page for *A. niger* Partially Purified Pectinase

**Fig. 7:**
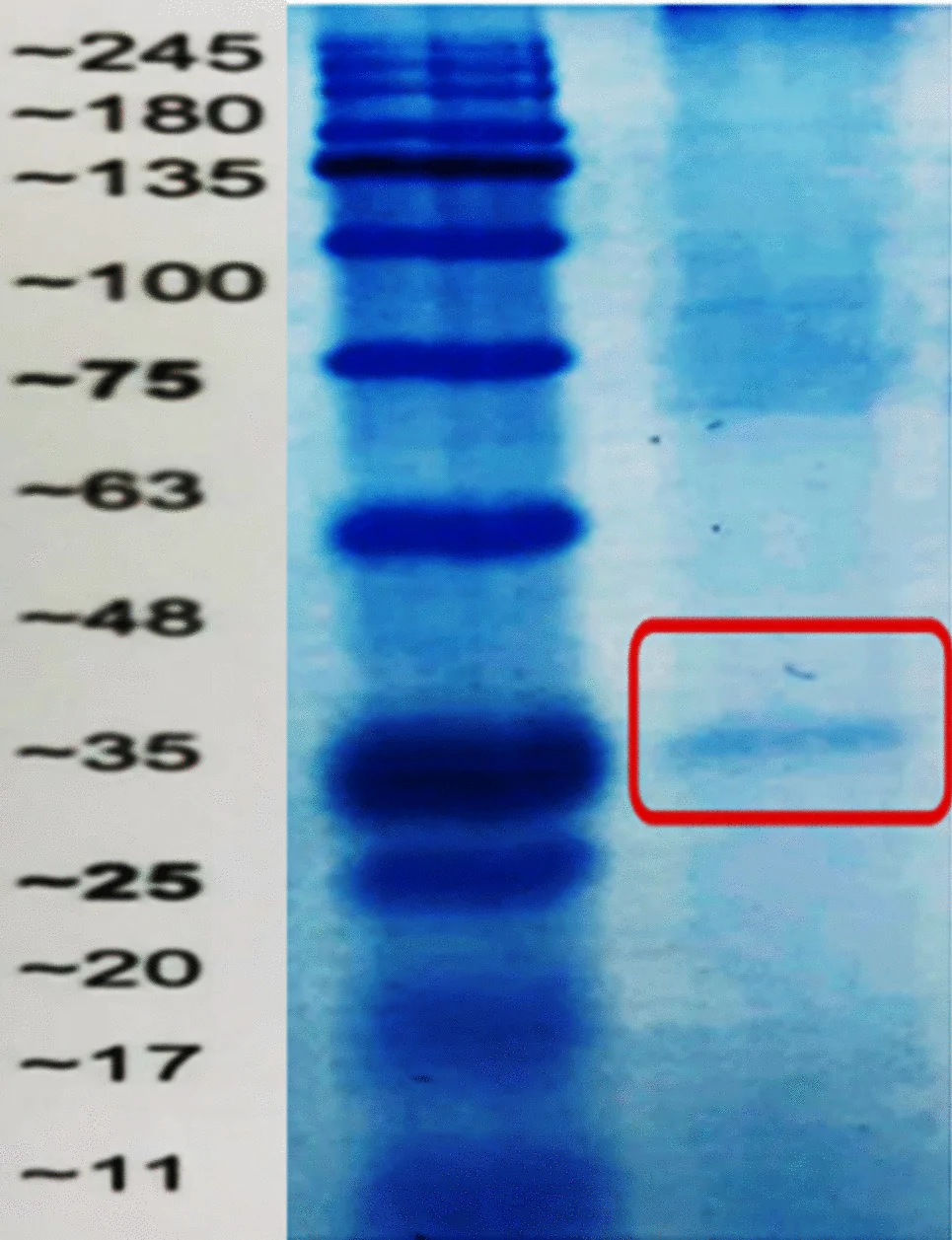
SDS-PAGE of partially purified pectinase from *A. niger* compared to M protein marker of Tris–glycine 4–20% in kilo Dalton (KDa)

The SDS-PGE analysis indicated that the apparent molecular weight of the pectinase enzyme, *A. niger*, was around 35 kDa, as seen in Fig. 7. The running gel also displayed highly pure patterns.

## Biocompatibility of Pectinase

**Fig. 8:**
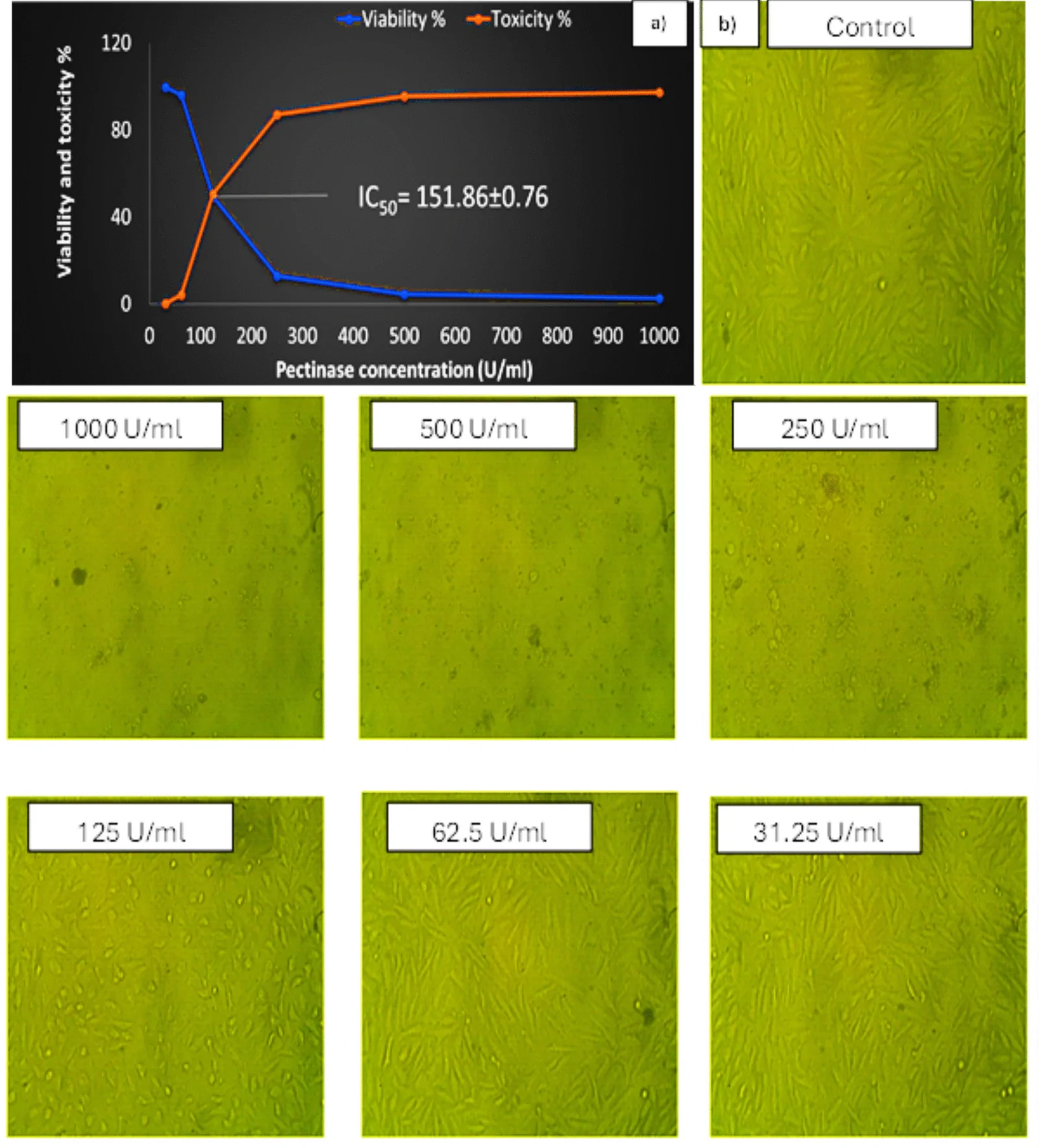
Cell viability affected by various concentrations of pectinase from *A. niger*. **a**) Human skin cell line (HFb-4) cells viability and IC_50_. **b**) Microscopic images for human skin cell line (HFb-4) before and after treatment with different concentrations of pectinase from *A. niger*

Before being used on a commercial scale, the biocompatibility of partially purified pectinase from *A. niger* against human skin cell line (HFb-4) was assessed. A medium degree of safety is indicated by the minimal half dosage (IC_50_), which was found to be 151.86 ± 0.76 U/ml (Fig. 8a). Large-scale alterations in cell surface morphology were noted, and the damage was recognized by significant volume reductions brought on by protein losses and intracellular ion losses as a result of altered permeability to sodium or potassium. Apoptotic cells exhibit nuclear fragmentation, nuclear condensation, and cell shrinkage, while necrotic cells exhibit nuclear swelling, chromatin flocculation, and lack of nuclear basophilia (Fig. 8b).

## Pectinase Commercial Application as Fabric Bioscouring Agent

**Fig. 9:**
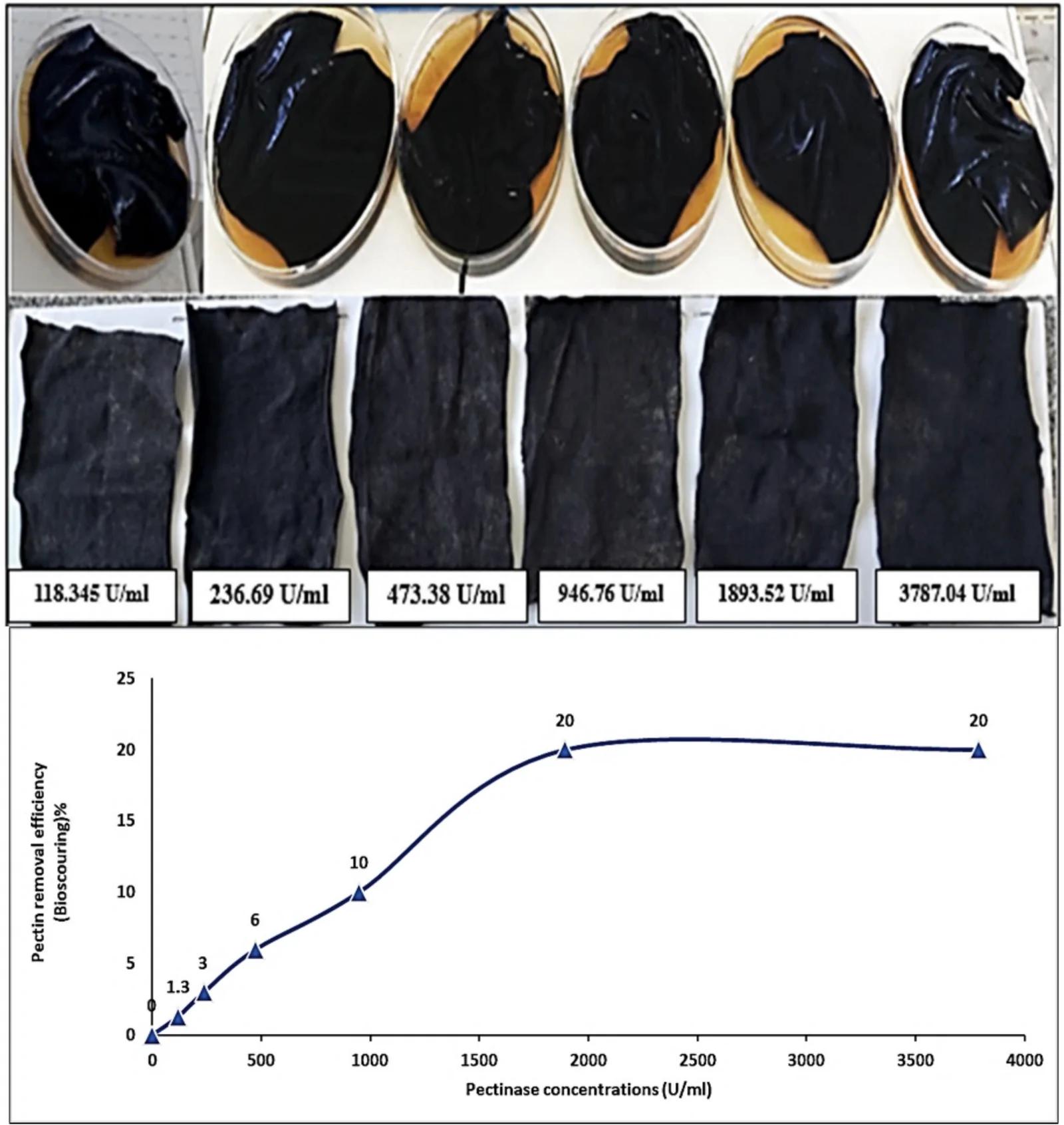
Bioscouring of cotton fabric with different concentrations of pectinase at 50 °C, and pH 7.0 for 45 min

Results illustrated in Fig. 9 clearly indicate that the bioscouring (removal of pectin) of the cloth greatly affected the different concentrations of partially purified pectinase enzyme, and 1893.52 U/ml gave the highest percentage of bioscouring being 20.0%. Further increase in pectinase concentration did not have any significant effect on the bioscouring of pectin in the fabric strip.

## Discussion

Of the commercial enzymes available worldwide, fungi account for 50%, bacteria for 35%, and plants or animals for the remaining 15%. The most common microorganisms used to produce pectinase are filamentous fungi [2, 33, 34]. In the breakdown of plant components, such as pectin, an essential part of plant cell walls, saprophytic fungi are essential. These fungi create pectinases, which are enzymes that break down pectin and help break down stiff plant tissues. They may recycle nutrients and support soil health thanks to their enzymatic activity. Fungi such as *Penicillium* sp., *Aspergillus* sp., and *Mucor* sp. are examples of pectinase-producing saprophytic fungi. The current study aimed to isolate fungi from different plants rhizosphere, in Fayoum, Egypt. Among 60, only 20 isolated fungi (33.35%) were discovered to be pectinase producers. Microscopic examination and colony morphology allowed for the isolates’ identification as *Aspergillus* sp. strains. Similar to this, several fungi isolated from soil samples in Manaslu Conservation Area, Gorkha [35, 36]. Eight isolates were found to be pectinase producers out of them; these were determined to be *Aspergillus niger* strains. Temperature, duration of incubation, and pectin (substrate) concentration all had a significant impact on enzyme activity. For this, it was discovered that pectinase was very active at pH 5.0, and 1% pectin after 5–7 days. In addition, pectinase activity was not significantly affected by an additional increase in substrate. In contrast to our findings, 2.5% pectin was found to be the best for maximizing pectinase activity [35, 37]. Also, *A. niger* produce pectinase after 4 days [36], whereas it was after 2 days of incubation [38]. However, after 5 days of incubation, *A. flavus* pectinase was most effectively generated [39]. Due to the fact that both the microbiological sources and the culture media’s composition affect pectinase synthesis at its best. A shorter fermentation cycle is advantageous for the industrial process from a business standpoint. Therefore, this separation may have industrial uses in addition to biological ones. The temperature was raised to 45–50 °C, after which the *A. niger* pectinase activity dropped, according to the obtained results. Pectinase that was produced from *A. niger* strain MCAS2 at temperature from 50 to 70 °C, with 50 °C being the ideal temperature [35, 36]. As the temperature rose, there was a little drop in the pectinase activity. At 100 °C, 82% of the pectinase activity was still present. This further demonstrates the thermostability of the pectinase enzyme from the *Aspergillus* sp. strains.

The ideal pH for *A. niger* pectinase activity is 5.0, while *P. virdicatum* pectinase is most active at pH values between 5.0 and 8.5 [35, 39–43]. In contrast, bacteria, particularly those belonging to the *Bacillus* species, are the primary producers of alkaline pectinase [44]. The pectinase enzyme from *B. pumilus* dcsr1, *B. stearothermophilus*, *P. xylanolyticus*, and *B. halondurans* M29 was active at high temperatures and pH levels [45]. The cysteine residue found in the amino acid sequence of pectinase may be responsible for this thermostability [46]. Cysteine residues have a high hydrophobic impact in addition to forming disulfide bonds, which contribute to thermostability [47]. The pectinase enzyme that was isolated from *A. niger* may include this residue, which gives the enzyme its thermostability. The most crucial properties of a biocatalyst for its usage in industrial applications are temperature and pH stability. This means that the fungal pectinase producer might be a more environmentally friendly option than the fungal thermostable alkaliphilic pectinase. It could also be useful for pretreating pectic effluent from the fruit juice industry, pulp and paper industries, and fiber crop degumming and retting.

The partial purification of crude pectinase from *A. niger* exhibited the highest relative activity at ammonium sulfate concentrations between 40 and 60% saturation. Pectinase was purified using the chilled acetone purification process, with specific activity of 8.33 U/ml [35, 39].

Fungal pectinase enzymes, particularly those from *Aspergillus* sp., have an active temperature range of 30–50 °C. However, they are inactive at temperatures above 50 °C due to denaturation. The optimal temperature for pectinase activity is 50 °C, while other enzymes remain active at 60 °C and 50–80 °C [39–42].

The partially purified pectinase from *A. niger* was found to be significantly active over a broad temperature range of 50–70 °C and pH 6.2–9.2, with an optimal temperature and pH of 50 °C, indicating thermostability and an alkaline nature of pectinase [35, 48]. Due to these characteristics, the enzyme is useful in a variety of industrial operations involving high pH and temperatures.

It was also discovered that the pectinase enzyme from *A. niger* had a molecular weight of about 35 kDa, while pectinase was purified resulting in an apparent molecular weight of 66 kDa [35, 42] and isolated 63 kDa pectinase from *Penicillium frequentans* [49]. In agreement with the present findings, pectinase molecular mass was in the range of 30–80 kDa [50].

Pectinase enzymes are a class of enzymes that break down pectin into polysaccharides, residues of galacturonic acid, and trace amounts of xylose, rhamnose, arabinose, and galactose [51]. The ultimate objective was to create a process for pectin’s full hydrolysis. Acetic acid ethyl ester, hexadecane carbonsaeure methylase, hexadecenoic acid, 9-octadecenoic acid (z), cis-vaccenic acid, octadecanoic acid, oleic acid, glycidyl palmitate, ethyl iso-alcoholate, 2,3-dihydroxypropyl ester, and arabinitol pentaacetate were the principal end products, according to the GC–MS analysis of the pectinase metabolites profile. Also, *A. aculeatus* pectinase was used to create a pectin hydrolysis profile that showed the amounts of galactose, arabinitol, neutral sugars, 3-dihydroxypropyl ester, rhamnose, mannose, and arabinose [52]. The final products of pectin degradation by gut microbiota were as follows: inositol, acetic acid, oleic acid, palmitic acid, stearic acid, petroselinic acid, pentadecanoic acid, monopalmitin, linoelaidic acid, and 10‐oxooctadecanoic acid [33].

The cytotoxic effects of pectinase against healthy African green monkey cells (VERO cells) and human colon cancer cells (Caco-2 cells) were studied [52]. Additionally, it was discovered to have an IC_50_ of 25 µg/ml with colon Caco-2 cells; on healthy VERO cells, however, its cytotoxic effects were less pronounced. The high cytotoxic effects on cancer cells could be potentiating in the anticancer agents.

The purpose of textile bioscouring is to get good hydrophilic characteristics and remove natural materials from textiles, such as pectin, lipids, and waxes. Large volumes of caustic soda and textile additives are needed for this old technique [53–55]. Moreover, it is necessary to neutralize the alkali that was utilized afterwards. Pectinase-based bioscouring is an alternative to high alkali application: in the bioscouring process, mildly alkaline textile auxiliaries are combined with enzymes, also known as biocatalysts. Pectinases aid in the removal of cotton’s interfering fiber components without the requirement for high treatment temperatures or significant alkali concentrations. Enzymatic breakdown makes it simple to extract the pectin from the cotton fiber’s main cell wall. Our multipurpose specialist products for bioscouring include wetting agents, surfactants, and enzyme compositions. These items offer exceptional process safety and are simple to use. Alkaline stable pectinase is employed in the process of “bioscouring,” which removes pectin and waxes from cotton fiber in a targeted manner. This technique is substrate specific and does not change the cellulose component, in contrast to conventional alkaline scouring [56, 57]. The study found that the bioscouring of cloth significantly influenced the concentration of partially purified pectinase enzyme, with 1893.52 U/ml achieving the highest bioscouring percentage of 20% at 50 °C after 45 min. Pectinase enzyme was isolated from *Fusarium* sp. and applied also in the textiles bioscouring agent [58]. Cotton fabric was distressed using the partially purified pectinase enzyme. They compared the bioscoured cotton fabric’s efficiency to that of the traditionally scoured fabric. The results showed that the bioscoured cloth had a much better water-absorbing capacity than the traditionally scoured fabric. Additionally, it was discovered that the cotton cloth treated with pectinase enzyme had a better tensile strength than the sample treated conventionally. Using 40% enzyme concentration, 4 h of treatment at 40 °C, and pH 5.0 were the optimal for the bioscouring of pre-treated cotton textiles using pectinase generated from the pectinolytic fungus *Paecilomyces variotii* [59].

## Conclusion

The study isolated 60 fungi isolates from five plant rhizospheres in Fayoum governorate, Egypt, to examine their pectinase production. The highest pectinase degrading index was scored for FB5, FJ2, and FW1 isolates. The most active pectinase-producing fungi were identified as *A. niveus* strain AUMC1624, *A. niger* strain AUMC16245, and *A. brasiliensis* strain AUMC16244. The fungi reached maximum thermostable pectinase levels at 1% pectin after 5, 7, and 7 days at 40, 45, and 45 °C, respectively. The study examined the thermostability of *A. niger* pectinase, revealing its increased activity with temperature and pH. The enzyme’s molecular weight was approximately 35 kDa. It showed biocompatibility effects and was applied as a clothes bioscouring agent. The results could be used in various sectors, including agriculture and food, for pesticides and juice production. So we might deduce that thermostable and neutral-alkaline pectinase from *A. niger* with excellent pH and temperature stability of this pectinase enzyme makes it useful for a wide range of industrial processes, such as wastewater treatment, cotton fabric processing in the textile industry, and fruit juice extraction and clarity.

## Supplementary Information

Below is the link to the electronic supplementary material.

Supplementary file1 (PDF 288 KB)

## Data Availability

The authors declare that the article contains all the data established and analyzed during this investigation. All microbial strains were deposited in the following strain providers 1-*Aspergillus niveus* strain AUMC16243 https://www.ncbi.nlm.nih.gov/nuccore/PP279730.1/ 2-*Aspergillus brasiliensis* strain AUMC16244 https://www.ncbi.nlm.nih.gov/nuccore/PP279840.1/ 3-*Aspergillus niger* strain AUMC16245 https://www.ncbi.nlm.nih.gov/nuccore/PP279841.1/.

## References

[1] AbdRahman NH, Rahman RA, Rahmat Z et al (2024) Innovative biocatalyst synthesis of pectinolytic enzymes by cross-linking strategy: potentially immobilised pectinases for the production of pectic-oligosaccharides from pectin. Inter J Biolog Macromol 256:128260. 10.1016/j.ijbiomac.2023.128260 10.1016/j.ijbiomac.2023.128260 38000618

[2] Blanco-Pérez F, Steigerwald H, Schülke S et al (2021) The dietary fiber pectin: health benefits and potential for the treatment of allergies by modulation of gut microbiota. Curr Allerg Asthma Rep 21(10). 10.1007/s11882-021-01020-z 10.1007/s11882-021-01020-z PMC8433104 34505973

[3] Kavuthodi B, Sebastian D (2018) Review on bacterial production of alkaline pectinase with special emphasis on Bacillus species. Biosci Biotech Res Commun 11:18–30. 10.21786/bbrc/11.1/4

[4] Beukema M, Faas MM, De Vos P (2020) The effects of different dietary fiber pectin structures on the gastrointestinal immune barrier: impact via gut microbiota and direct effects on immune cells. Exper Mol Med 52(9):1364–1376. 10.1038/s12276-020-0449-2 32908213 10.1038/s12276-020-0449-2 PMC8080816

[5] Mondal S, Halder SK, Mondal KC (2024) Recombinant fungal pectinase and their role towards fostering modern agriculture. In Entrepreneurship with Microorganisms (pp 405–418). 10.1016/b978-0-443-19049-0.00003-7

[6] de Alencar Guimarães NC, Glienke NN, Contato AG et al (2024) Production and biochemical characterization of Aspergillus japonicus pectinase using a low-cost alternative carbon source for application in the clarification of fruit juices. Waste Bio Valor 15(1):177–186. 10.1007/s12649-023-02171-y

[7] Satpathy A, Mukherjee K, Nigam VK (2023) Batch cultivation and optimization of pectinase production using Bacillus sp.(BIOSMNF02) through RSM-D-optimal quadratic model. Biom Conver Bioref 1–12. 10.1007/s13399-023-04915-1

[8] Miranda PH, Morais RA, Sousa H et al (2024) Effects of pectinase treatment on the optimization and extraction of pigments from bacupari, tucumã, and peach palm using response surface methodology. J Braz Chem Soci 35:e20230124. 10.21577/0103-5053.20230124

[9] Laswai FC, Matofari JW, Nduko JM (2023) Pectinolytic enzyme production from orange processing waste using *Aspergillus brasiliensis* strain. Bio Conv Bioref 1–14. 10.1007/s13399-023-04603-0

[10] Li J, Peng C, Mao A et al (2024) An overview of microbial enzymatic approaches for pectin degradation. Inter J Biol Macromol 254:127804. 10.1016/j.ijbiomac.2023.127804 10.1016/j.ijbiomac.2023.127804 37913880

[11] Nisha MK (2016) Process optimization for bioscouring of cotton fabrics with pectinase obtained from *Paecilomyces variotii*. Int J Curr Microbiol App Sci 5(6):292–299. 10.20546/ijcmas.2016.506.033

[12] Ketipally R, Ram MR (2018) Optimization of pectinase production by *Aspergillus oryzae* RR 103. Curr Agric Res J 6(1):37. 10.12944/CARJ.6.1.05

[13] Abd-Elhalem BT, El-Sawy M, Gamal RF et al (2015) Production of amylases from *Bacillus amyloliquefaciens* under submerged fermentation using some agro-industrial by-products. Ann Agric Sci 60(2):193–202. 10.1016/j.aoas.2015.06.001

[14] Gupta A, Dhakan DB, Maji A et al (2019) Association of *Flavonifractor plautii*, a flavonoid-degrading bacterium, with the gut microbiome of colorectal cancer patients in India. MSystems 4(6):10–1128. 10.1128/mSystems.00438-19 10.1128/mSystems.00438-19 PMC7407896 31719139

[15] Barnett HL, Hunter BB (1972) Illustrated genera of imperfect fungi, 3rd edn. Burgess Publishing Co., Minneapolis, p 241

[16] Sambrook J, Fritsch EF, Maniatis T (1989) Molecular cloning: a laboratory manual, 2nd edn. Cold Spring Harbor Laboratory Press, New York

[17] Ahmed A, Khan MN, Ahmad A et al (2019) Optimization of pectinase production from Geotrichum candidum AA15 using response surface methodology. Pakistan J Bot 51:743–750

[18] Miller GL (1959) Use of dinitrosalicylic acid reagent for determination of reducing sugar. Anal Chem 31(3):426–428

[19] Ashwini K, Kumar G, Karthik L et al (2011) Optimization, production and partial purification of extracellular α-amylase from *Bacillus* sp marini. Arch Appl Sci Res 3:33–42

[20] Abd-Elhalem BT (2015) Production of an-extracellular starch degrading enzyme(s) by some bacteria. Thesis Ain Shams University, Faculty of Agriculture, Cairo

[21] Abd-Elhalem BT, Gamal RF, Abu-Hussien SH et al (2023) Optimizing alpha-amylase from *Bacillus amyloliquefaciens* on bread waste for effective industrial wastewater treatment and textile desizing through response surface methodology. Scie Rep 13(1):1–17. 10.1038/s41598-023-46384-6 10.1038/s41598-023-46384-6 PMC10628158 37932353

[22] Hamilton LM, Kelly CT, Fogarty WM (1999) Purification and properties of the raw starch degrading α-amylase of *Bacillus* sp. IMD434. Biotechnol Lett 21:111–115

[23] Fossi BT, Tavea F, Jiwoua C et al (2011) Simultaneous production of raw starch degrading highly thermostable α-amylase and lactic acid by *Lactobacillus fermentum* 04BBA19. Afr J Biotech 10:6564–6574

[24] EF Hartree 1972 Determination of protein: A modification of the lowery method that gives a linear photometric response Anal Biochem 48 422 427 T4. Nature, 227(5259), 680–685 10.1038/227680a0 10.1016/0003-2697(72)90094-2 4115981

[25] Maalej HH, Ben A, Olfa G et al (2014) Production and biochemical characterization of a high maltotetraose (G4) producing amylase from *Pseudomonas stutzeri* AS22. Biomed Res Int 43:499–510. 10.1155/2014/156438 10.1155/2014/156438 PMC4055539 24963472

[26] Uzyol KS, Akbulut BS, Denizci AA et al (2012) Thermostable α-amylase from moderately halophilic Halomonas sp AAD21. Turk J Biol 36:1–12. 10.3906/biy-1106-7

[27] Vrancheva RZ, Dincheva IN, Aneva IY et al (2020) Metabolite profiling by means of GC-MS combined with principal component analyses of natural populations of *Nectaroscordum siculum* ssp. bulgaricum (Janka) Stearn. Z Nat C 75:451–457. 10.1515/znc-2020-0058 10.1515/znc-2020-0058 32706756

[28] Laemmli UK (1970) Cleavage of structural proteins during the assembly of the head of bacteriophage T4. Nature 227(5259):680–685. 10.1038/227680a0 5432063 10.1038/227680a0

[29] van de Loosdrecht AA, Beelen RH, Ossenkoppele GJ et al (1994) A tetrazolium-based colorimetric MTT assay to quantitate human monocyte mediated cytotoxicity against leukemic cells from cell lines and patients with acute myeloid leukemia. J Immunol meth 174(1–2):311–320. 10.1016/0022-1759(94)90034-5 10.1016/0022-1759(94)90034-5 8083535

[30] Mojsov K (2012) Biotechnological applications of pectinases in textile processing and bioscouring of cotton fibers. In: II International Conference Industrial Engineering and Environmental Protection (IIZS 2012), Proceedings, Zrenjanin, Serbia. pp 314–322

[31] Duncan DB (1955) Multiple range and multiple F test. Biomet 11:1–42

[32] Claire V, Gregory JZ (2001) Hyperthermophilic enzymes: Sources, uses, and molecular mechanisms for thermostability. Microbiol Mol Biol Rev 65:1–43 11238984 10.1128/MMBR.65.1.1-43.2001 PMC99017

[33] Huang W, Fang Q, Fan L et al (2022) Pectin with various degrees of esterification differentially alters gut microbiota and metabolome of healthy adults. EFood 3(1–2):e5. 10.1002/efd2.5

[34] Alqahtani YS, More SS, Shaikh RK et al (2022). Production and purification of pectinase from *Bacillus subtilis* 15A-B92 and its biotechnological applications. Mol 27(13). 10.3390/molecules27134195 10.3390/molecules27134195 PMC9268039 35807437

[35] Khatri BP, Bhattarai T, Shrestha S et al (2015) Alkaline thermostable pectinase enzyme from *Aspergillus niger* strain MCAS2 isolated from Manaslu Conservation Area, Gorkha. Nepal SpringerPlus 4:488. 10.1186/s40064-015-1286-y 26380164 10.1186/s40064-015-1286-y PMC4564381

[36] Dwivedi S, Kanchan Y, Supriya G et al (2023) Fungal pectinases: an insight into production, innovations and applications. World J Microb Biotech 39. 10.1007/s11274-023-03741-x 10.1007/s11274-023-03741-x 37691054

[37] Carrasco M, Rozas JM, Alcaíno J (2019) Pectinase secreted by psychrotolerant fungi: identification, molecular characterization and heterologous expression of a cold-active polygalacturonase from *Tetracladium* sp. Microb Cell Fact 18:45. 10.1186/s12934-019-1092-2 30845994 10.1186/s12934-019-1092-2 PMC6407229

[38] Tripathi GD, Javed Z, Sushma A (2014) Pectinase production and purification from *Bacillus subtilis* isolated from soil. Adv Appl Sci Res 5(1):103–105

[39] Martin N, Guez MAU, Sette LD et al (2010) Pectinase production by a Brazilian thermophilic fungus *Thermomucor indicae-seudaticae* N31 in solid-state and submerged fermentation. Mikrobiol 79(3):321–328. 10.1134/S0026261710030057 20734812

[40] Gewali MB, Maharjan J, Thapa S et al (2007) Studies on polygalacturonase from *Aspergillus flavus*. Sci World 5(5):19–22. 10.3126/sw.v5i5.2650

[41] Banu AR, Devi MK, Ganaprabhal GR et al (2010) Production and characterization of pectinase from *Penicillium chrysogenum*. Indian J Sci Technol 3(4):377–381. 10.17485/ijst/2010/v3i4/29721

[42] Maciel MDC, Herculano PN, Porto TS et al (2011) Production and partial characterization of pectinases from forage palm by *Aspergillus niger* URM4645. Afr J Biotechnol 10(13):2469–2475

[43] Fahmy AS, El-Beih FM, Mohamed SA et al (2008) Characterization of an exopolygalacturonase from *Aspergillus niger*. Appl Biochem Biotech 149(3):205–217. 10.1007/s12010-007-8107-x 10.1007/s12010-007-8107-x 18500582

[44] Kumar A, Sharma R (2012) Production of alkaline pectinase by bacteria (Cocci sps) isolated from decomposting fruit materials. J Phytol 4(1):1–5

[45] Giacobbe S, Pepe O, Ventorino V et al (2014) Identification and characterization of a pectinolytic enzyme from *Paenibacillus xylanolyticus*. Biores 9(3):4873–4887. 10.15376/biores.9.3.4873-4887

[46] Singh R, Dhawan S, Singh K et al (2012) Cloning, expression and characterization of a metagenome derived thermoactive/thermostable pectinase. Mol biol rep 39(8):8353–8361. 10.1007/s11033-012-1685-x 22711301 10.1007/s11033-012-1685-x

[47] You C, Huang Q, Xue H et al (2010) Potential hydrophobic interaction between two cysteines in interior hydrophobic region improves thermostability of a family 11 xylanase from *Neocallimastix patriciarum*. Biotech Bioeng 105(5):861–870. 10.1002/bit.22623 10.1002/bit.22623 19998284

[48] Mat JMT, Ibrahim D (2021) Partial purification and characterisation of pectinase produced by *Aspergillus niger* LFP-1 grown on pomelo peels as a substrate. Trop Life Sci Res 32(1):1–22. 10.21315/tlsr2021.32.1.1 10.21315/tlsr2021.32.1.1 PMC8054668 33936548

[49] Barense RI, Chellegatti MASC, Fonseca MJV et al (2001) Partial purification and characterization of exopolygalacturonase II and III of *Penicillium frequentans*. Braz J Microbiol 32:327–330. 10.1590/S1517-83822001000400014

[50] de Vries RP, Visser J (2001) *Aspergillus* enzymes involved in degradation of plant cell wall polysaccharides. MMBR 65(4):497–522. 10.1128/MMBR.65.4.497-522.2001 11729262 10.1128/MMBR.65.4.497-522.2001 PMC99039

[51] Bang SJ, Kim G, Lim MY (2018) The influence of in vitro pectin fermentation on the human fecal microbiome. AMB Expr 8:98. 10.1186/s13568-018-0629-9 10.1186/s13568-018-0629-9 PMC6004267 29909506

[52] Wikiera A, Mika M, Starzyńska-Janiszewska A et al (2015) Development of complete hydrolysis of pectins from apple pomace. Food Chem 172:675–680. 10.1016/j.foodchem.2014.09.132 25442606 10.1016/j.foodchem.2014.09.132

[53] Almeida EA, Facchi SP, Martins AF et al (2015) Synthesis and characterization of pectin derivative with antitumor property against Caco-2 colon cancer cells. Carb Poly 115:139–145. 10.1016/j.carbpol.2014.08.085 10.1016/j.carbpol.2014.08.085 25439878

[54] Puranen T, Alapuranen M, Vehmaanperä J (2013) Trichoderma enzymes for textile industries. Biotech Biol of Tricho 351–362. 10.1016/B978-0-444-59576-8.00026-6

[55] Zhang S, Bilal M, Zdarta J et al (2021) Biopolymers and nanostructured materials to develop pectinases-based immobilized nano-biocatalytic systems for biotechnological applications. Food Res Int 140:109979. 10.1016/j.foodres.2020.109979 33648214 10.1016/j.foodres.2020.109979

[56] Joshi S, Satyanarayana T (2014) In vitro engineering of microbial enzymes with multifarious applications: prospects and perspectives. Biores Technol 176:273–283. 10.1016/j.biortech.2014.10.151 10.1016/j.biortech.2014.10.151 25435065

[57] Singh A, Bajar S, Devi A et al (2021) An overview on the recent developments in fungal cellulase production and their industrial applications. Biore Technol Repo 14:100652. 10.1016/j.biteb.2021.100652

[58] Rajendran R, Sundaram SK, Radhai R et al (2011) Bioscouring of cotton fabrics using pectinase enzyme its optimization and comparison with conventional scouring process. Pak J Biol Sci 14(9):519–525. 10.3923/pjbs.2011.519.525 22032080 10.3923/pjbs.2011.519.525

[59] Silva D, Martins ESD, Silva RD et al (2002) Pectinase production by *Penicillium viridicatum* RFC3 by solid state fermentation using agricultural wastes and agro-industrial by-products. Braz J Microbiol 33:318–324. 10.1590/S1517-83822002000400008

