# Supplementary material for: Study of Different Cultivated Plants Rhizosphere Soil Fungi-Mediated Pectinase: Insights into Production, Optimization, Purification, Biocompatibility, and Application
Source: Microb Ecol. 2025 Jan 6;87:165. doi: 10.1007/s00248-024-02474-0 (PMC11703994; doi:10.1007/s00248-024-02474-0)
Supplement: Supplementary file 1 [file 248_2024_2474_MOESM1_ESM.pdf]

Supplementary material

Journal name: Microbial Ecology

Manuscript Title: Insights on pectinase production by soil fungi: Optimization, purification, biocompatibility, and application.

Authors information

1- Mai Ali Mwaheb
Affiliation: Botany Department, Faculty of Science, Fayoum University, Fayoum 63514, Egypt.
E-mail: mam08@fayoum.edu.eg
Phone:+0201006974633
ORCID: 0000-0002-0128-387X

2- Basant Mohamed Abd El- Aziz
Affiliation: Botany Department, Faculty of Science, Fayoum University, Fayoum 63514, Egypt.
E-mail: bma12@fayoum.edu.eg
Phone:+020106959104

3- Basma T. Abd-Elhalim* (Corresponding author)
Affiliation: Department of Agricultural Microbiology, Faculty of Agriculture, Ain Shams University, Shubra El-Khaimah, Cairo 11241, Egypt.
E-mail: basma.talaat@agr.asu.edu.eg
ORCID: 0000-0002-3009-332X
Phone number:+201007025808

4- Nabil Abo El-Kassim
Affiliation: Botany Department, Faculty of Science, Fayoum University, Fayoum 63514, Egypt.
E-mail: naa05@fayoum.edu.eg
Phone: +20106 908 3939

5- Tharwat E.E. Radwan
Affiliation: Botany Department, Faculty of Science, Fayoum University, Fayoum 63514, Egypt.
E-mail: tsd00@fayoum.edu.eg
Phone: +20 111 537 8355

Table S1. Total number and percentage of fungal isolates obtained from different plants rhizosphere.

Isolation source	Number of total isolates	Pectinase producing isolates	Pectinase non-producing isolates	Isolates codes
**Banana (*Musa acuminata*)**	22	6	16	FB1
				FB2
				FB3
				FB4
				FB5
				FB6
**Jarawa (*Glossonema varians*)**	6	6	0	FJ1
				FJ2
				FJ3
				FJ4
				FJ5
				FJ6
**Lemon (*Citrus aurantiifolia*)**	10	2	8	FL1
				FL2
**Tomato (*Solanum lycopersicum*)**	18	3	15	FT1
				FT2
				FT3
**Wheat (*Triticum aestivum* L.)**	4	3	1	FW1
				FW2
				FW3
**Total number**	60	20	40	
**Percentage %**	100%	33.3%	66.7%	

Table S2. Qualitative and quantitative screening for pectinases producing fungi isolates.

Isolation source	Isolate code	CD (cm)	IZD (cm)	PDI %	PA (U/ml)
**Banana** ***(Musa acuminata)***	FB1	2.70^{de}	1.50^{g}	80.0	1245.0^{c}
	FB2	4.80^{c}	3.15^{e}	52.4	566.81^{f}
	FB3	7.83^{ab}	6.38^{c}	22.7	280.62^{i}
	FB4	8.93^{a}	8.83^{a}	1.13	25.700^{o}
	FB5	8.90^{a}	4.90^{d}	81.6	1603.7^{a}
	FB6	8.10^{a}	7.03^{b}	15.2	586.10^{f}
**Jarawa** ***(Glossonema varians)***	FJ1	2.88^{de}	1.00^{g}	88.3	1311.2^{b}
	FJ2	4.50^{c}	2.90^{e,f}	55.2	1236.7^{cd}
	FJ3	2.70^{de}	1.50^{g}	80.0	14.231^{p}
	FJ4	4.07^{c}	3.53^{e}	15.1	330.66^{h}
	FJ5	4.37^{c}	2.43^{f}	79.5	1092.3^{d}
	FJ6	6.70^{b}	5.63^{c}	18.9	456.81^{g}
**Lemon** ***(Citrus aurantiifolia)***	FL1	4.50^{c}	3.47^{e}	29.8	55.175^{n}
	FL2	2.72^{de}	1.50^{g}	81.1	156.39^{k}
**Tomato** ***(Solanum lycopersicum)***	FT1	1.83^{f}	1.08^{g}	69.2	59.331^{n}
	FT2	3.23^{d}	2.37^{f}	36.6	93.736^{l}
	FT3	8.47^{a}	7.50^{b}	12.9	83.132^{lm}
**Wheat** **(*Triticum aestivum* L.)**	FW1	1.50^{f}	0.75^{h}	100	264.82^{h}
	FW2	8.33^{a}	6.00^{c}	38.9	257.53^{ij}
	FW3	3.95^{cd}	2.40^{f}	64.6	700.78^{e}

CD= Colony diameter, IZD=Inhibition zone diameter, PDI=Pectin degradation index, PA=Pectinase activity. Values in the same column followed by the same letter are not significantly different, according to Duncan at a 5% level.

Table S3. Partial purification of fungal pectinase by different *Aspergillus* spp. using ammonium sulphate and organic solvent methods.

Purification method	*A. brasiliensis*			
	Activity	Protein	Specific activity	Relative activity %
Acetone	1189.97[c]	1.670[e]	712.56	31.42
Ethanol	3489.21[b]	34.50[b]	101.14	92.14
Ammonium sulphate 20%	3578.35[a]	11.65[c]	307.15	94.42
Ammonium sulphate 40%	3878.35[a]	109.1[a]	32.77	94.49
Ammonium sulphate 60%	3496.40[b]	7.460[d]	468.69	92.33
Purification method	*A. niger*			
	Activity	Protein	Specific activity	% Relative activity
Acetone	3475.76[d]	18.5[d]	187.88	89.62
Ethanol	2333.02[e]	0.43[e]	114.19	60.15
Ammonium sulphate 20%	3493.12[b]	202.5[a]	17.25	90.07
Ammonium sulphate 40%	3787.04[a]	76.61[b]	48.13	95.07
Ammonium sulphate 60%	3459.96[bc]	51.56[c]	67.11	89.21
Purification method	*A. niveus*			
	Activity	Protein	Specific activity	Relative activity
Acetone	3572.95[a]	13.47[d]	250.40	94.40
Ethanol	2120.8[de]	12.12[de]	174.98	59.36
Ammonium sulphate 20%	2188.99[d]	47.05[b]	46.52	61.27
Ammonium sulphate 40%	2882.57[c]	65.53[a]	43.98	80.68
Ammonium sulphate 60%	3294.04[b]	40.06[c]	82.22	92.20

Table S4. Temperature coefficient (Q10) for pectinase by *A. niger***.**

**Temperature °C**	**k1**	**k2**	**Q10**
30-40	1905.35	2133.25	1.12
40-50	2133.25	3088.42	1.45
50-60	3088.42	2821.99	0.91
60-70	2821.99	2558.72	0.91

Table S5. GC-MS analysis of pectinase end products profile of by *A. niger*.

Retention Time (min)	Area%	Compound Name	Formula
5.04	0.09	Acetic acid ethyl ester	$C_4H_8O_2$
15.16	0.14	1,2-Bis(2-hydroxyphenyl) ethylenediamine N,N'-bis(3-ethylbut-2-en-1-ylidene)	$C_{24}H_{28}N_2O_2$
15.16	0.14	N,n'-Bis-(3-methyl-but-2-enylidene)-1,2-di-2-hydroxyphenyl-ethane-1,2-diamine	$C_{24}H_{28}N_2O_2$
32.02	0.24	1,4-Benzenediol, 2-(1,1-dimethylethyl)-5-(2-propenyl)	$C_{13}H_{18}O_2$
32.02	0.24	3,4-Dihydro-2h-1,5-(3''-t-utyl) benzodiazepine	$C_{13}H_{18}O_2$
32.02	0.24	15-Methyltricyclo[6.5.2(13,14).0(7 ,15)]pentadeca-1,3,5,7,9,11,13-heptene	$C_{16}H_{14}$
41.17	0.25	Docosane	$C_{22}H_{46}$
40.04	0.18	Dotriacontane	$C_{32}H_{66}$
40.42	0.09	3-OXO-20-methyl-11-à-hydroxyconanine-1,4-diene	$C_{22}H_{31}NO_2$
43.53	0.46	4A,5,8,8Aà-Tetrahydro-2-methoxy-4aá,8à-dimethyl-1,4-naphthalindione	$C_{13}H_{16}O_3$
43.89	0.11	4H-1-Benzopyran-4-one,2-(3,4 dihydroxyphenyl)-6,8-di-á-d-glucopyranosyl-5,7-dihydroxy	$C_{27}H_{30}O_{16}$
43.89	0.11	Phthalic acid, butyl undecyl ester	$C_{23}H_{36}O_4$
45.40	0.74	7,9-di-tert-butyl-1-oxaspiro[4.5]deca-6,9-diene-2,8-dione	$C_{17}H_{24}O_3$
45.89	0.25	2-Tert-butyl-3,3,7,7-tetramethyl-7h-tetrahydroindeno[bc]furan	$C_{18}H_{28}O$
45.89	0.25	1-Amino-3,4-dihydro-3-methyl-4-phenyl-2-naphthalene carbonitrile	$C_{18}H_{16}N_2$
46.77	5.73	Hexadecane carbonsaeuremethylese	$C_{17}H_{34}O_2$
48.19	6.71	Hexadecenoic acid	$C_{16}H_{32}O_2$
48.72	0.20	Hexadecenoic acid, 2,3-dihydroxypropyl ester	$C_{19}H_{38}O_4$
50.54	0.10	3,20-Dioxo-11-à-hydroxyconanine-1,4-diene	$C_{21}H_{27}NO_3$
51.40	0.10	Dotriacontane	$C_{32}H_{66}$
51.40	0.10	Isochiapin b	$C_{19}H_{22}O_6$
52.95	1.71	Methyl stearate /octadecanoic acid, methyl ester	$C_{19}H_{38}O_2$
52.65	4.99	9-Octadecenoic acid (z)	$C_{19}H_{36}O$
53.65	4.99	Cis-Vaccenic acid	$C_{18}H_{34}O_2$
54.02	0.34	Hi-Oleic safflower oil	$C_{21}H_{22}O_{11}$
54.33	4.95	Octadecanoic acid	$C_{18}H_{36}O_2$
58.27	1.39	Glycidyl palmitate	$C_{19}H_{36}O_3$
58.97	0.14	Oleic acid	$C_{18}H_{34}O_2$
59.87	0.35	Cyclopropane butanoic acid, 2-[[2-[[2-[(2-pentylcyclopropyl)methyl]cyclopropyl]methyl]cyclopropyl]methyl]-, methyl ester	$C_{25}H_{42}O_2$

60.46	0.42	Methyl8-(5-hexyl-2-hienyl) octanoate	$C_{19}H_{32}O_2S$
61.12	0.11	7-Methyl-Z-tetradecen-1-ol acetate	$C_{17}H_{32}O_2$
62.04	0.36	9,12-Octadecadienoic acid (Z,Z)-, 2-hydroxy-1-(hydroxymethyl)ethyl ester	$C_{21}H_{38}O_4$
62.62	0.17	12-Methyl-E,E-2,13-octadecadien-1-ol	$C_{19}H_{36}O$
63.77	2.40	9-Octadecenoic acid(Z)-, oxiranylmethyl ester	$C_{21}H_{38}O_3$
64.70	1.19	Hexadecenoic acid,3-[(trimethylsilyl)oxy]propyl ester	$C_{22}H_{46}O_3Si$
64.98	0.10	Ethyliso-allocholate	$C_{26}H_{44}O_5$
65.61	0.26	Isochiapin	$C_{19}H_{22}O_6$
65.95	0.17	Cyclopropane butanoic acid,2-[[2-[[2-[(2-pentyl cyclopropyl)methyl]cyclopropyl]methyl]cyclopropyl] methyl]-,methyl ester	$C_{25}H_{42}O_2$
69.40	0.34	2-hydroxy-3-[(9e)-9-octadecenoyloxy]propyl(9e)-9-octadecenoate #	$C_{39}H_{72}O_5$
69.80	0.79	9,12,15-Octadecatrienoic acid,2-[(trimethyls lyl)oxy]-[[(trimethylsilyl)oxy]methyl]ethyl ester, (z,z,z)	$C_{27}H_{52}O_4Si_2$
69.80	0.79	9-Octadecenoic acid(Z)-, oxiranylmethyl	$C_{21}H_{38}O_3$
69.80	0.79	9,10-Secocholesta-5,7,10(19)-triene-1,3-diol, 25-[(trimethylsilyl)oxy]-,(3á,5z,7e)	$C_{30}H_{52}O_3Si$
70.22	3.54	Octadecanoic acid, 2,3-dihydroxypropyl ester	$C_{21}H_{42}O_4$
71.21	0.20	4H-1-Benzopyran -4-one, 2-(3,4-dihydroxyphenyl)-6,8-di-á-d-glucopyranosyl-5,7-dihydroxy	$C_{27}H_{30}O_{16}$
77.37	0.14	Ethyl iso-alcoholate	$C_{26}H_{44}O_5$
78.72	0.24	Trilinolein	$C_{57}H_{98}O_6$
81.53	0.08	Arabinitol, pentaacetate	$C_{15}H_{22}O_{10}$
81.94	0.36	2-Hydroxy-3-[(9e)-9-octadecenoyloxy]propyl(9e)-9-octadecenoate	$C_{39}H_{72}O_5$
83.12	0.37	9,12-Octadecadienoic acid(z,z)-, 2,3-bis[(trimethylsilyl)oxy]propyl ester	$C_{27}H_{54}O_4Si_2$
87.67	0.20	Antirliine	$C_{19}H_{24}N_2O$
91.59	3.85	5,11,17,23-Tetrakis(1,1-dimethylethyl)-28-methoxypentacyclo[19.3.1.1(3,7).1(9,13).1(15,19)]octacosa-1(25),3,5,7(28),9,11,13(27),15,17,19(26),21,23-dodecene-25,26,27-triol	$C_{45}H_{58}O_4$
