# Supplementary material for: Study of Different Cultivated Plants Rhizosphere Soil Fungi-Mediated Pectinase: Insights into Production, Optimization, Purification, Biocompatibility, and Application
Source: Microb Ecol. 2025 Jan 6;87(1):165. doi: 10.1007/s00248-024-02474-0 (PMC11703994; doi:10.1007/s00248-024-02474-0)
Supplement: Supplementary file 1 — Supplementary file1 (PDF 288 KB) [file 248_2024_2474_MOESM1_ESM.pdf]

# Supplementary material

**Journal name:** Microbial Ecology

**Manuscript Title:** Insights on pectinase production by soil fungi: Optimization, purification, biocompatibility, and application.

**Authors information**

**1- Mai Ali Mwaheb**
Affiliation: Botany Department, Faculty of Science, Fayoum University, Fayoum 63514, Egypt.
E-mail: mam08@fayoum.edu.eg
Phone:+0201006974633
ORCID: 0000-0002-0128-387X

**2- Basant Mohamed Abd El- Aziz**
Affiliation: Botany Department, Faculty of Science, Fayoum University, Fayoum 63514, Egypt.
E-mail: bma12@fayoum.edu.eg
Phone:+020106959104

**3- Basma T. Abd-Elhalim* (Corresponding author)**
Affiliation: Department of Agricultural Microbiology, Faculty of Agriculture, Ain Shams University, Shubra El-Khaimah, Cairo 11241, Egypt.
E-mail: basma.talaat@agr.asu.edu.eg
ORCID: 0000-0002-3009-332X
Phone number:+201007025808

**4- Nabil Abo El-Kassim**
Affiliation: Botany Department, Faculty of Science, Fayoum University, Fayoum 63514, Egypt.
E-mail: naa05@fayoum.edu.eg
Phone: +20106 908 3939

**5- Tharwat E.E. Radwan**
Affiliation: Botany Department, Faculty of Science, Fayoum University, Fayoum 63514, Egypt.
E-mail: tsd00@fayoum.edu.eg
Phone: +20 111 537 8355

**Table S1.** Total number and percentage of fungal isolates obtained from different plants rhizosphere.

| Isolation source | Number of total isolates | Pectinase producing isolates | Pectinase non-producing isolates | Isolates codes |
|---|---|---|---|---|
| **Banana (*Musa acuminata*)** | 22 | 6 | 16 | FB1 |
| | | | | FB2 |
| | | | | FB3 |
| | | | | FB4 |
| | | | | FB5 |
| | | | | FB6 |
| **Jarawa (*Glossonema varians*)** | 6 | 6 | 0 | FJ1 |
| | | | | FJ2 |
| | | | | FJ3 |
| | | | | FJ4 |
| | | | | FJ5 |
| | | | | FJ6 |
| **Lemon (*Citrus aurantiifolia*)** | 10 | 2 | 8 | FL1 |
| | | | | FL2 |
| **Tomato (*Solanum lycopersicum*)** | 18 | 3 | 15 | FT1 |
| | | | | FT2 |
| | | | | FT3 |
| **Wheat (*Triticum aestivum* L.)** | 4 | 3 | 1 | FW1 |
| | | | | FW2 |
| | | | | FW3 |
| **Total number** | 60 | 20 | 40 | |
| **Percentage %** | 100% | 33.3% | 66.7% | |

**Table S2.** Qualitative and quantitative screening for pectinases producing fungi isolates.

| Isolation source | Isolate code | CD (cm) | IZD (cm) | PDI % | PA (U/ml) |
|---|---|---|---|---|---|
| **Banana** ***(Musa acuminata)*** | FB1 | $2.70^{de}$ | $1.50^{g}$ | 80.0 | $1245.0^{c}$ |
| | FB2 | $4.80^{c}$ | $3.15^{e}$ | 52.4 | $566.81^{f}$ |
| | FB3 | $7.83^{ab}$ | $6.38^{c}$ | 22.7 | $280.62^{i}$ |
| | FB4 | $8.93^{a}$ | $8.83^{a}$ | 1.13 | $25.700^{o}$ |
| | FB5 | $8.90^{a}$ | $4.90^{d}$ | 81.6 | $1603.7^{a}$ |
| | FB6 | $8.10^{a}$ | $7.03^{b}$ | 15.2 | $586.10^{f}$ |
| **Jarawa** ***(Glossonema varians)*** | FJ1 | $2.88^{de}$ | $1.00^{g}$ | 88.3 | $1311.2^{b}$ |
| | FJ2 | $4.50^{c}$ | $2.90^{e,f}$ | 55.2 | $1236.7^{cd}$ |
| | FJ3 | $2.70^{de}$ | $1.50^{g}$ | 80.0 | $14.231^{p}$ |
| | FJ4 | $4.07^{c}$ | $3.53^{e}$ | 15.1 | $330.66^{h}$ |
| | FJ5 | $4.37^{c}$ | $2.43^{f}$ | 79.5 | $1092.3^{d}$ |
| | FJ6 | $6.70^{b}$ | $5.63^{c}$ | 18.9 | $456.81^{g}$ |
| **Lemon** ***(Citrus aurantiifolia)*** | FL1 | $4.50^{c}$ | $3.47^{e}$ | 29.8 | $55.175^{n}$ |
| | FL2 | $2.72^{de}$ | $1.50^{g}$ | 81.1 | $156.39^{k}$ |
| **Tomato** ***(Solanum lycopersicum)*** | FT1 | $1.83^{f}$ | $1.08^{g}$ | 69.2 | $59.331^{n}$ |
| | FT2 | $3.23^{d}$ | $2.37^{f}$ | 36.6 | $93.736^{l}$ |
| | FT3 | $8.47^{a}$ | $7.50^{b}$ | 12.9 | $83.132^{lm}$ |
| **Wheat** **(*Triticum aestivum* L.)** | FW1 | $1.50^{f}$ | $0.75^{h}$ | 100 | $264.82^{h}$ |
| | FW2 | $8.33^{a}$ | $6.00^{c}$ | 38.9 | $257.53^{ij}$ |
| | FW3 | $3.95^{cd}$ | $2.40^{f}$ | 64.6 | $700.78^{e}$ |

CD= Colony diameter, IZD=Inhibition zone diameter, PDI=Pectin degradation index, PA=Pectinase activity. Values in the same column followed by the same letter are not significantly different, according to Duncan at a 5% level.

**Table S3.** Partial purification of fungal pectinase by different *Aspergillus* spp. using ammonium sulphate and organic solvent methods.

| Purification method | *A. brasiliensis* | | | |
|---|---|---|---|---|
| | Activity | Protein | Specific activity | Relative activity % |
| Acetone | 1189.97[c] | 1.670[e] | 712.56 | 31.42 |
| Ethanol | 3489.21[b] | 34.50[b] | 101.14 | 92.14 |
| Ammonium sulphate 20% | 3578.35[a] | 11.65[c] | 307.15 | 94.42 |
| Ammonium sulphate 40% | 3878.35[a] | 109.1[a] | 32.77 | 94.49 |
| Ammonium sulphate 60% | 3496.40[b] | 7.460[d] | 468.69 | 92.33 |
| Purification method | *A. niger* | | | |
| | Activity | Protein | Specific activity | % Relative activity |
| Acetone | 3475.76[d] | 18.5[d] | 187.88 | 89.62 |
| Ethanol | 2333.02[e] | 0.43[e] | 114.19 | 60.15 |
| Ammonium sulphate 20% | 3493.12[b] | 202.5[a] | 17.25 | 90.07 |
| Ammonium sulphate 40% | 3787.04[a] | 76.61[b] | 48.13 | 95.07 |
| Ammonium sulphate 60% | 3459.96[bc] | 51.56[c] | 67.11 | 89.21 |
| Purification method | *A. niveus* | | | |
| | Activity | Protein | Specific activity | Relative activity |
| Acetone | 3572.95[a] | 13.47[d] | 250.40 | 94.40 |
| Ethanol | 2120.8[de] | 12.12[de] | 174.98 | 59.36 |
| Ammonium sulphate 20% | 2188.99[d] | 47.05[b] | 46.52 | 61.27 |
| Ammonium sulphate 40% | 2882.57[c] | 65.53[a] | 43.98 | 80.68 |
| Ammonium sulphate 60% | 3294.04[b] | 40.06[c] | 82.22 | 92.20 |

**Table S4.** Temperature coefficient (Q10) for pectinase by *A. niger*.

| **Temperature °C** | **k1** | **k2** | **Q10** |
|---|---|---|---|
| 30-40 | 1905.35 | 2133.25 | 1.12 |
| 40-50 | 2133.25 | 3088.42 | 1.45 |
| 50-60 | 3088.42 | 2821.99 | 0.91 |
| 60-70 | 2821.99 | 2558.72 | 0.91 |

**Table S5.** GC-MS analysis of pectinase end products profile of by *A. niger*.

| Retention Time (min) | Area% | Compound Name | Formula |
|---|---|---|---|
| 5.04 | 0.09 | Acetic acid ethyl ester | $C_4H_8O_2$ |
| 15.16 | 0.14 | 1,2-Bis(2-hydroxyphenyl) ethylenediamine N,N'-bis(3-ethylbut-2-en-1-ylidene) | $C_{24}H_{28}N_2O_2$ |
| 15.16 | 0.14 | N,n'-Bis-(3-methyl-but-2-enylidene)-1,2-di-2-hydroxyphenyl-ethane-1,2-diamine | $C_{24}H_{28}N_2O_2$ |
| 32.02 | 0.24 | 1,4-Benzenediol, 2-(1,1-dimethylethyl)-5-(2-propenyl) | $C_{13}H_{18}O_2$ |
| 32.02 | 0.24 | 3,4-Dihydro-2h-1,5-(3"-t-utyl) benzodiazepine | $C_{13}H_{18}O_2$ |
| 32.02 | 0.24 | 15-Methyltricyclo[6.5.2(13,14).0(7 ,15)]pentadeca-1,3,5,7,9,11,13-heptene | $C_{16}H_{14}$ |
| 41.17 | 0.25 | Docosane | $C_{22}H_{46}$ |
| 40.04 | 0.18 | Dotriacontane | $C_{32}H_{66}$ |
| 40.42 | 0.09 | 3-OXO-20-methyl-11-à-hydroxyconanine-1,4-diene | $C_{22}H_{31}NO_2$ |
| 43.53 | 0.46 | 4A,5,8,8Aà-Tetrahydro-2-methoxy-4aá,8à-dimethyl-1,4-naphthalindione | $C_{13}H_{16}O_3$ |
| 43.89 | 0.11 | 4H-1-Benzopyran-4-one,2-(3,4 dihydroxyphenyl)-6,8-di-á-d-glucopyranosyl-5,7-dihydroxy | $C_{27}H_{30}O_{16}$ |
| 43.89 | 0.11 | Phthalic acid, butyl undecyl ester | $C_{23}H_{36}O_4$ |
| 45.40 | 0.74 | 7,9-di-tert-butyl-1-oxaspiro[4.5]deca-6,9-diene-2,8-dione | $C_{17}H_{24}O_3$ |
| 45.89 | 0.25 | 2-Tert-butyl-3,3,7,7-tetramethyl-7h-tetrahydroindeno[bc]furan | $C_{18}H_{28}O$ |
| 45.89 | 0.25 | 1-Amino-3,4-dihydro-3-methyl-4-phenyl-2-naphthalene carbonitrile | $C_{18}H_{16}N_2$ |
| 46.77 | 5.73 | Hexadecane carbonsaeuremethylese | $C_{17}H_{34}O_2$ |
| 48.19 | 6.71 | Hexadecenoic acid | $C_{16}H_{32}O_2$ |
| 48.72 | 0.20 | Hexadecenoic acid, 2,3-dihydroxypropyl ester | $C_{19}H_{38}O_4$ |
| 50.54 | 0.10 | 3,20-Dioxo-11-à-hydroxyconanine-1,4-diene | $C_{21}H_{27}NO_3$ |
| 51.40 | 0.10 | Dotriacontane | $C_{32}H_{66}$ |
| 51.40 | 0.10 | Isochiapin b | $C_{19}H_{22}O_6$ |
| 52.95 | 1.71 | Methyl stearate /octadecanoic acid, methyl ester | $C_{19}H_{38}O_2$ |
| 52.65 | 4.99 | 9-Octadecenoic acid (z) | $C_{19}H_{36}O$ |
| 53.65 | 4.99 | Cis-Vaccenic acid | $C_{18}H_{34}O_2$ |
| 54.02 | 0.34 | Hi-Oleic safflower oil | $C_{21}H_{22}O_{11}$ |
| 54.33 | 4.95 | Octadecanoic acid | $C_{18}H_{36}O_2$ |
| 58.27 | 1.39 | Glycidyl palmitate | $C_{19}H_{36}O_3$ |
| 58.97 | 0.14 | Oleic acid | $C_{18}H_{34}O_2$ |
| 59.87 | 0.35 | Cyclopropane butanoic acid, 2-[[2-[[2-[(2-pentylcyclopropyl)methyl]cyclopropyl]methyl]cyclopropyl]methyl]-, methyl ester | $C_{25}H_{42}O_2$ |

| | | | |
|---|---|---|---|
| 60.46 | 0.42 | Methyl8-(5-hexyl-2-hienyl) octanoate | $C_{19}H_{32}O_2S$ |
| 61.12 | 0.11 | 7-Methyl-Z-tetradecen-1-ol acetate | $C_{17}H_{32}O_2$ |
| 62.04 | 0.36 | 9,12-Octadecadienoic acid (Z,Z)-, 2-hydroxy-1-(hydroxymethyl)ethyl ester | $C_{21}H_{38}O_4$ |
| 62.62 | 0.17 | 12-Methyl-E,E-2,13-octadecadien-1-ol | $C_{19}H_{36}O$ |
| 63.77 | 2.40 | 9-Octadecenoic acid(Z)-, oxiranylmethyl ester | $C_{21}H_{38}O_3$ |
| 64.70 | 1.19 | Hexadecenoic acid,3-[(trimethylsilyl)oxy]propyl ester | $C_{22}H_{46}O_3Si$ |
| 64.98 | 0.10 | Ethyliso-allocholate | $C_{26}H_{44}O_5$ |
| 65.61 | 0.26 | Isochiapin | $C_{19}H_{22}O_6$ |
| 65.95 | 0.17 | Cyclopropane butanoic acid,2-[[2-[[2-[(2-pentyl cyclopropyl)methyl]cyclopropyl]methyl]cyclopropyl] methyl]-,methyl ester | $C_{25}H_{42}O_2$ |
| 69.40 | 0.34 | 2-hydroxy-3-[(9e)-9-octadecenoyloxy]propyl(9e)-9-octadecenoate # | $C_{39}H_{72}O_5$ |
| 69.80 | 0.79 | 9,12,15-Octadecatrienoic acid,2-[(trimethylslyl)oxy]-[[(trimethylsilyl)oxy]methyl]ethyl ester, (z,z,z) | $C_{27}H_{52}O_4Si_2$ |
| 69.80 | 0.79 | 9-Octadecenoic acid(Z)-, oxiranylmethyl | $C_{21}H_{38}O_3$ |
| 69.80 | 0.79 | 9,10-Secocholesta-5,7,10(19)-triene-1,3-diol, 25-[(trimethylsilyl)oxy]-,(3á,5z,7e) | $C_{30}H_{52}O_3Si$ |
| 70.22 | 3.54 | Octadecanoic acid, 2,3-dihydroxypropyl ester | $C_{21}H_{42}O_4$ |
| 71.21 | 0.20 | 4H-1-Benzopyran -4-one, 2-(3,4-dihydroxyphenyl)-6,8-di-á-d-glucopyranosyl-5,7-dihydroxy | $C_{27}H_{30}O_{16}$ |
| 77.37 | 0.14 | Ethyl iso-alcoholate | $C_{26}H_{44}O_5$ |
| 78.72 | 0.24 | Trilinolein | $C_{57}H_{98}O_6$ |
| 81.53 | 0.08 | Arabinitol, pentaacetate | $C_{15}H_{22}O_{10}$ |
| 81.94 | 0.36 | 2-Hydroxy-3-[(9e)-9-octadecenoyloxy]propyl(9e)-9-octadecenoate | $C_{39}H_{72}O_5$ |
| 83.12 | 0.37 | 9,12-Octadecadienoic acid(z,z)-, 2,3-bis[(trimethylsilyl)oxy]propyl ester | $C_{27}H_{54}O_4Si_2$ |
| 87.67 | 0.20 | Antirliine | $C_{19}H_{24}N_2O$ |
| 91.59 | 3.85 | 5,11,17,23-Tetrakis(1,1-dimethylethyl)-28-methoxypentacyclo[19.3.1.1(3,7).1(9,13).1(15,19)]octacosa-1(25),3,5,7(28),9,11,13(27),15,17,19(26),21,23-dodecene-25,26,27-triol | $C_{45}H_{58}O_4$ |
